# Supplementary material for: Structural titration reveals Ca2+-dependent conformational landscape of the IP3 receptor
Source: Nat Commun. 2023 Oct 28;14:6897. doi: 10.1038/s41467-023-42707-3 (PMC10613215; doi:10.1038/s41467-023-42707-3)
Supplement: Supplementary file 1 — Supplementary Information [file 41467_2023_42707_MOESM1_ESM.pdf]

Structural titration reveals Ca^{2+}-dependent conformational landscape of the IP_3 receptor

Navid Paknejad*[1,2], Vinay Sapuru*[1,2], Richard K. Hite#[1]

[1] Structural Biology Program, Memorial Sloan Kettering Cancer Center, New York, NY, 10065

[2] Physiology, Biophysics, and Systems Biology (PBSB) Program, Weill Cornell Graduate School of Biomedical Sciences, 1300 York Avenue, New York, NY, 10065, USA

* Equal contribution

# Correspondence to hiter@mskcc.org

This PDF file includes:

Supplementary Figures 1-18

Supplementary Tables 1-12

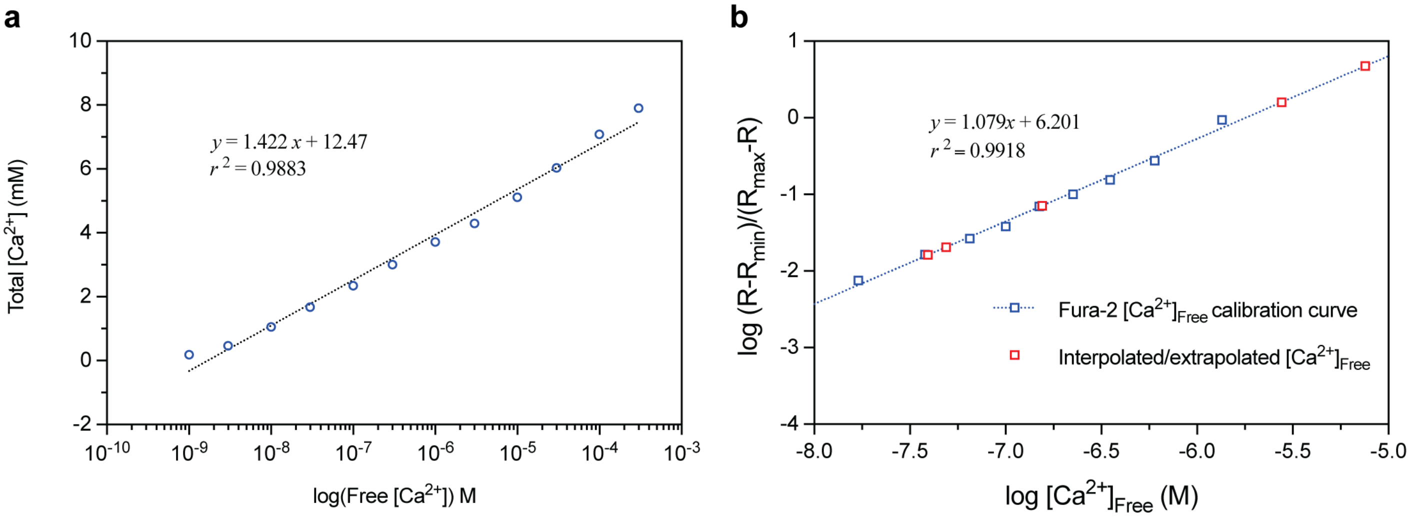

c

WEBMAXC estimated $[Ca^{2+}]_{Free}$ (nM)	Fura-2 interpolated/ extrapolated $[Ca^{2+}]_{Free}$ (nM)
1	39.35
10	48.97
100	155.07
1000	2770.45
10000	7582.85

Supplementary Figure 1: Modeling and experimental assessment of the Ca^{2+} buffering system. **a,** Modeling of free versus total Ca^{2+} in the experimental buffer using MaxChelator. Points were fit to a line with r^2=0.99. **b,** The Fura-2 Ca^{2+} calibration curve (dotted blue line) was generated by fitting a straight line through the emission ratio values of samples from the Invitrogen Calcium Calibration Buffer Kit (blue squares). Red squares represent interpolated (10^0, 10^1,10^2 nM) and extrapolated (10^3, 10^4 nM) values of free Ca^{2+} in the ligand and Ca^{2+} chelator cocktail. **c,** Interpolated/extrapolated values of free Ca^{2+} in the ligand and Ca^{2+} chelator cocktail, determined based on the Fura-2 calibration curve. Source data are provided as a Source Data file.

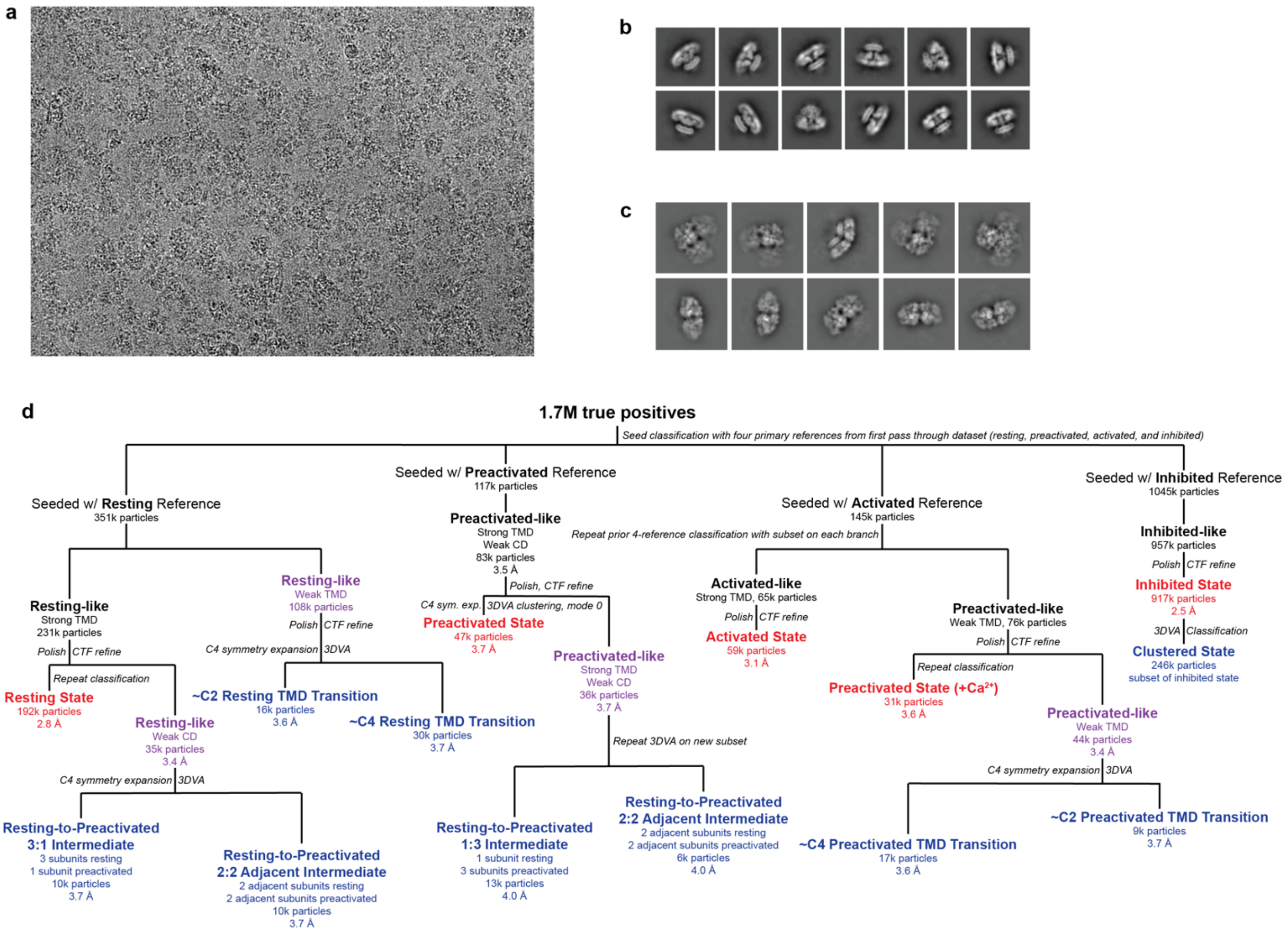

Supplementary Figure 2: Cryo-EM classification workflow highlighting key observations and decision-making criteria. a, Representative cryo-EM micrograph of the 17,688 collected micrographs in the merged data set. **b-c,** Representative 2D classification of merged particles (b) and higher-order inhibited state particles (c). **d,** 3D classification workflow for merged particles. When appropriate, particle numbers are presented along with $FSC_{0.143}$ refinement resolution. States depicted in red are the primary C4 symmetric states. Classes depicted in purple are conformational ensembles that were investigated using 3DVA. States depicted in blue are conformational states obtained from 3DVA that are derived from the preceding ensemble.

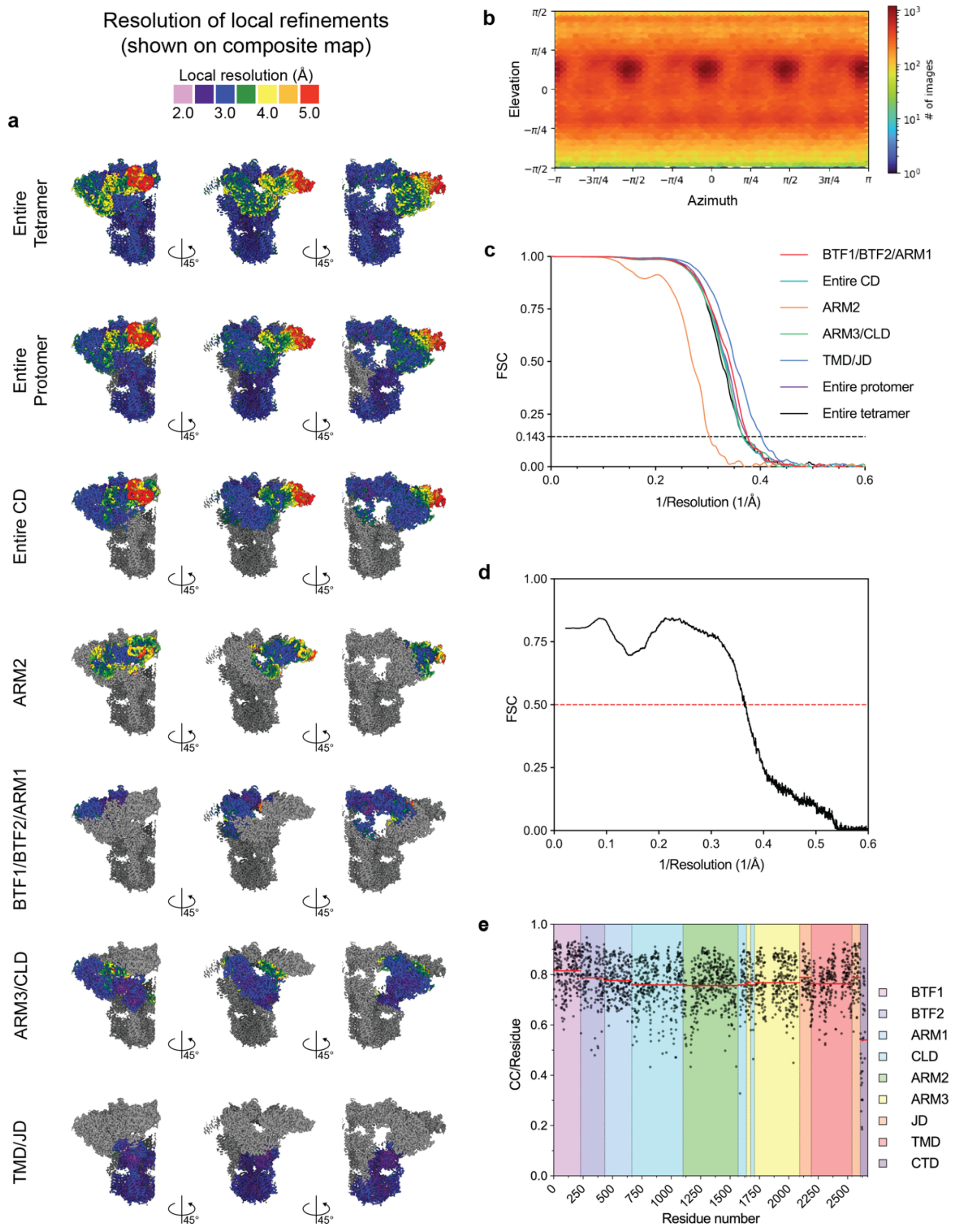

Supplementary Figure 3: Validation of the resting state. a, Local resolution plots at $FSC_{0.5}$ for consensus and local refinements depicted on the final density modified composite map. **b,** Angular distribution plot for the consensus refinement. **c,** Half map $FSC_{0.143}$ plots for all refinements. **d,** $FSC_{0.5}$ map versus model. **e,** Per-residue cross correlation (CC) for map-to-model comparison with domain demarcations and a red bar highlighting the average CC for each domain.

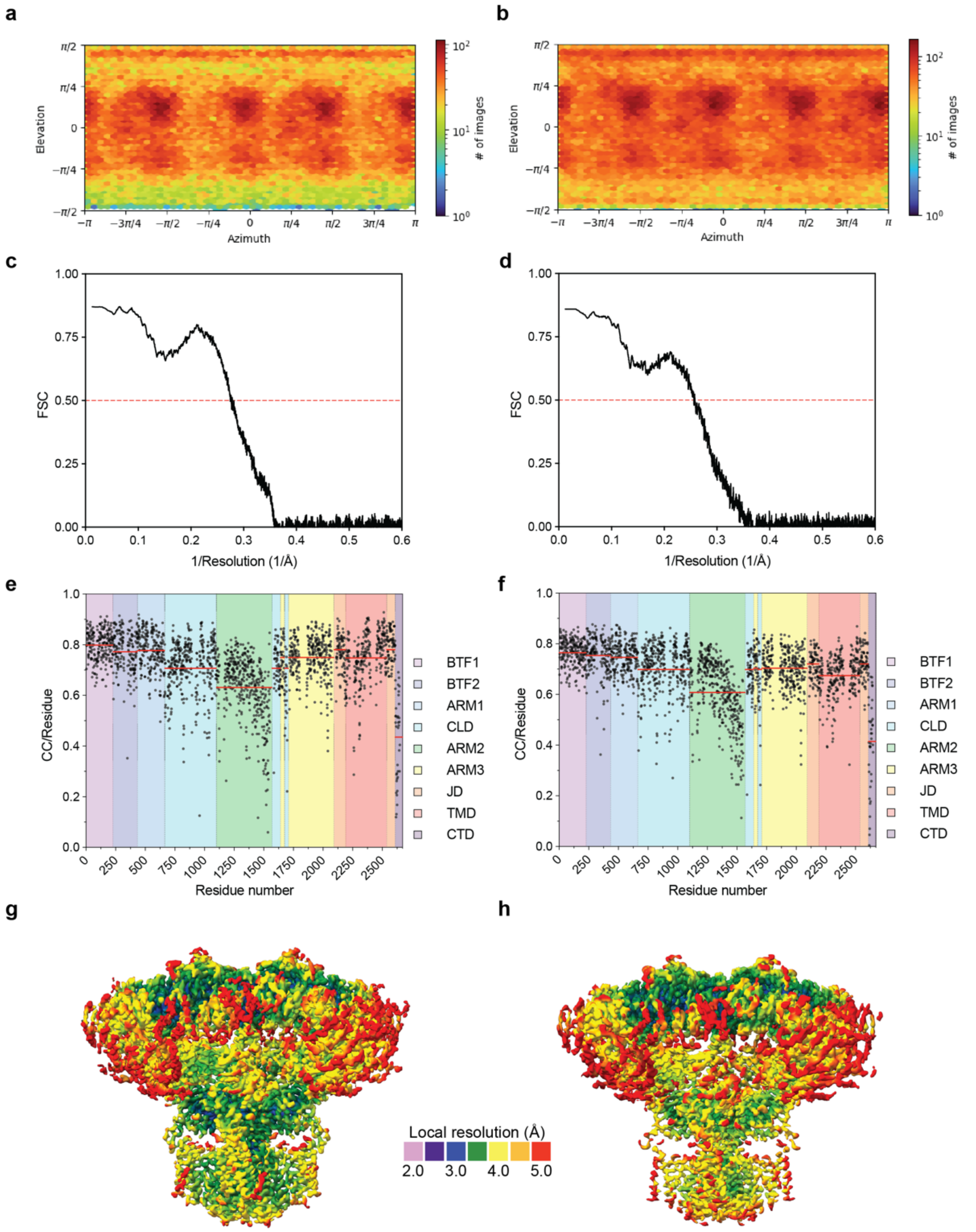

Supplementary Figure 4: Validation of the labile resting states. a-b, Angular distribution plot for the consensus refinement of (a) labile resting state #1 and (b) labile resting state #2. **c-d,** $FSC_{0.5}$ map versus model for (c) labile resting state #1 and (d) labile resting state #2. **e-f,** Per-residue cross correlation (CC) for map-to-model comparison with domain demarcations and a red bar highlighting the average CC for each domain for (e) labile resting state #1 and of (f) labile resting state #2. **g-h,** Local resolution plots at $FSC_{0.5}$ for specific consensus refinements depicted on the final density modified composite map of (g) labile resting state #1 and (h) labile resting state #2.

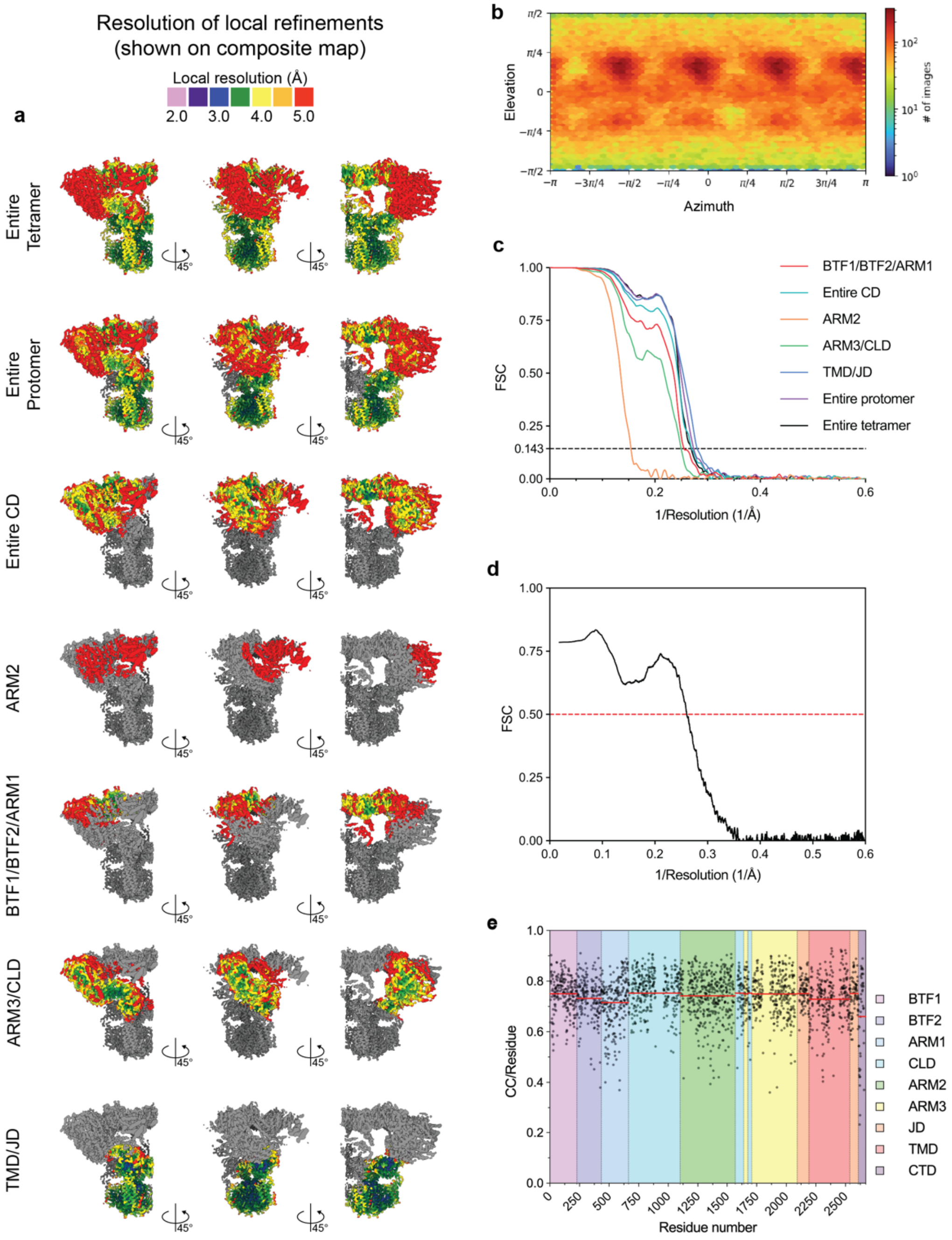

Supplementary Figure 5: Validation of the preactivated state. a, Local resolution plots at $FSC_{0.5}$ for consensus or local refinements depicted on the final density modified composite map. **b,** Angular distribution plot for the consensus refinement. **c,** Half map $FSC_{0.143}$ plots for all refinements. **d,** $FSC_{0.5}$ map versus model. **e,** Per-residue cross correlation (CC) for map-to-model comparison with domain demarcations and a red bar highlighting the average CC for each domain.

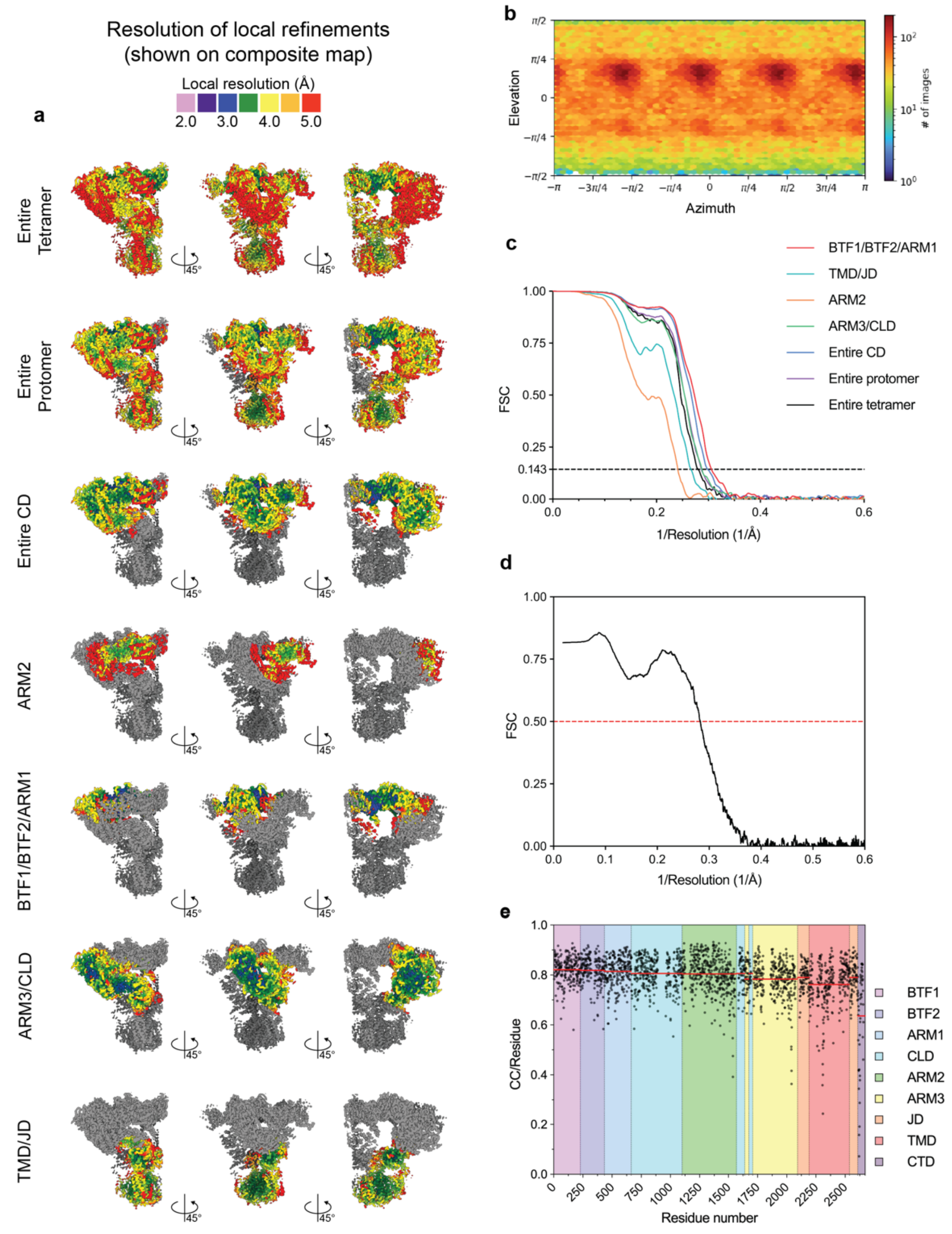

Supplementary Figure 6: Validation of the preactivated+Ca^{2+} state. a, Local resolution plots at $FSC_{0.5}$ for consensus or local refinements depicted on the final density modified composite map. **b,** Angular distribution plot for the consensus refinement. **c,** Half map $FSC_{0.143}$ plots for all refinements. **d,** $FSC_{0.5}$ map versus model. **e,** Per-residue cross correlation (CC) for map-to-model comparison with domain demarcations and a red bar highlighting the average CC for each domain.

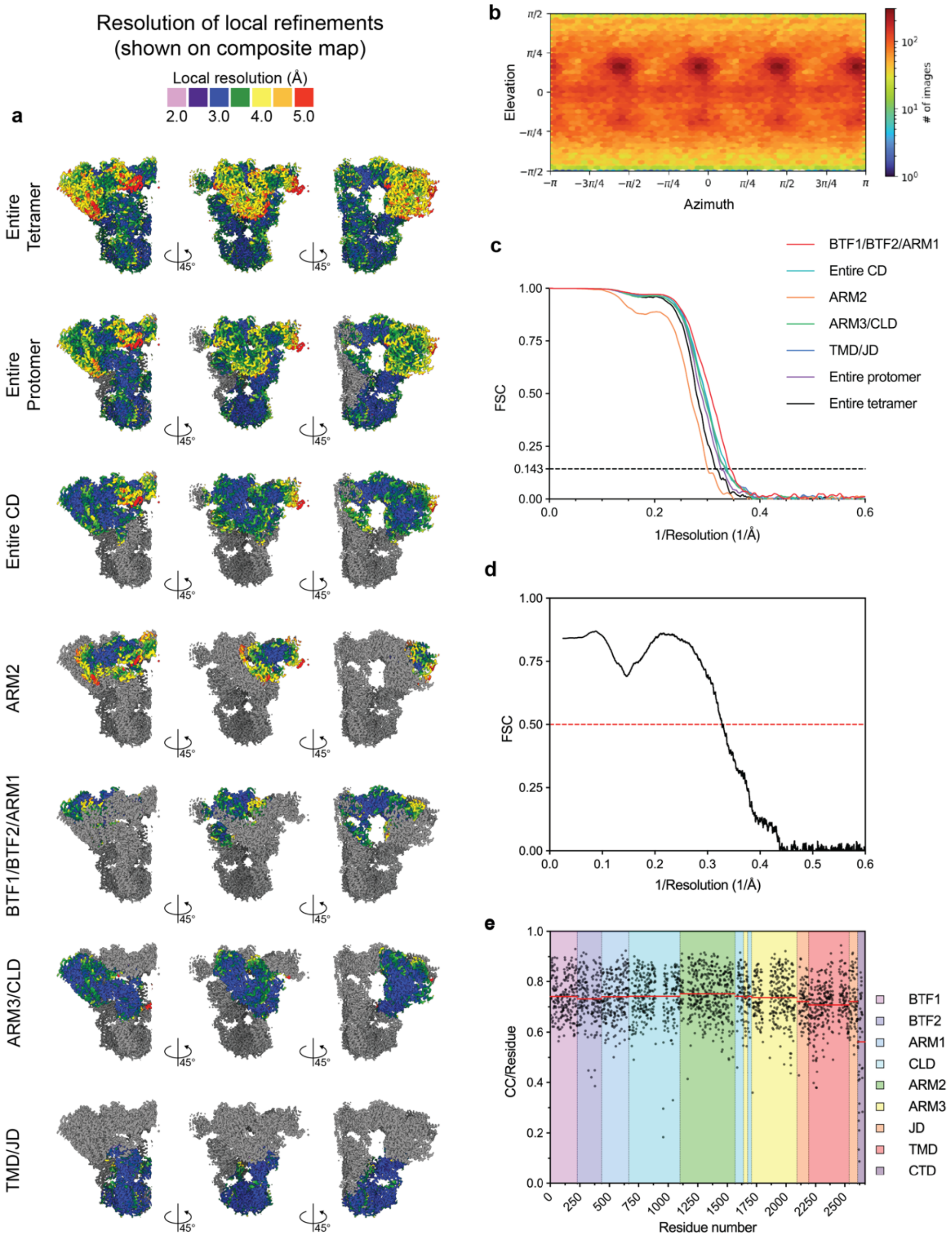

Supplementary Figure 7: Validation of the activated state. a, Local resolution plots at $FSC_{0.5}$ for consensus or local refinements depicted on the final density modified composite map. **b,** Angular distribution plot for the consensus refinement. **c,** Half map $FSC_{0.143}$ plots for all refinements. **d,** $FSC_{0.5}$ map versus model. **e,** Per-residue cross correlation (CC) for map-to-model comparison with domain demarcations and a red bar highlighting the average CC for each domain.

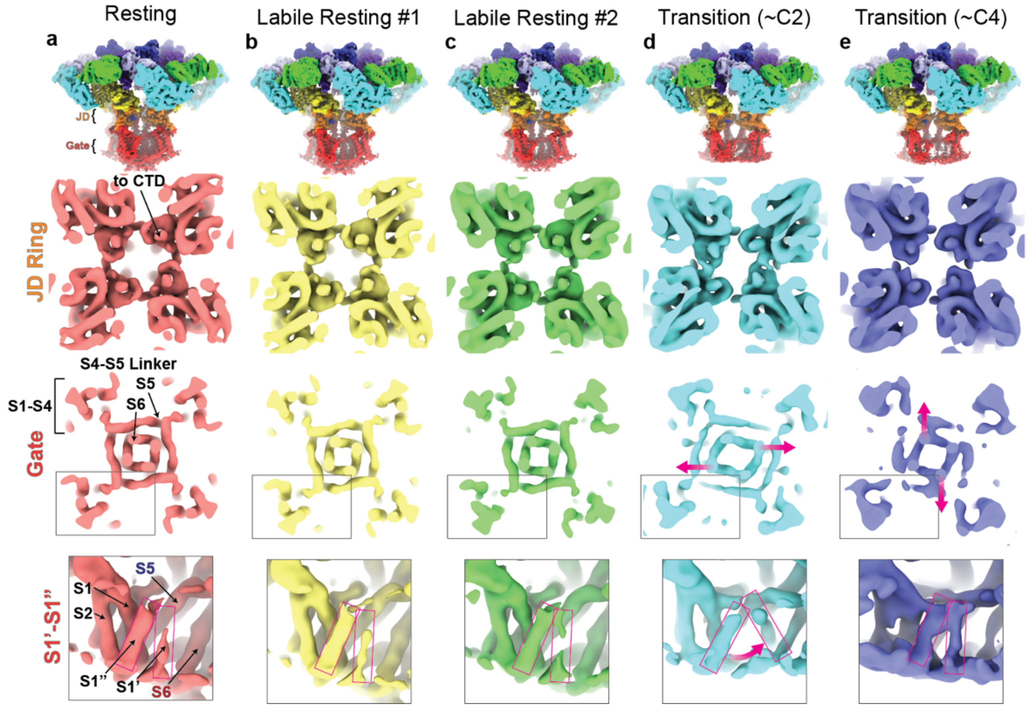

Supplementary Figure 8: Snapshots of conformational heterogeneity in the JD ring intact resting-like states. a-e, Cryo-EM density maps of the resting state (a), labile resting state #1 (b), labile resting state #2 (c), ~C2 resting TMD transition state (d), and ~C4 resting TMD transition state (e), low-pass filtered to 4 Å (overall) or 7 Å (slices). Row 1: Overall cryo-EM density. Row 2: density slice looking from the cytosol at the height of the JD ring. Row 3: density slice looking from the cytosol at the height of the gate. Row 4: Side view of a single S1-S4 domain highlighting the position of S1'-S1" and the S4-S5 linker for the central protomer, with S5 from the adjacent protomer and S6 of the opposite protomer visible, highlighting the intertwined TMD of domain-swapped 6TM cation channels.

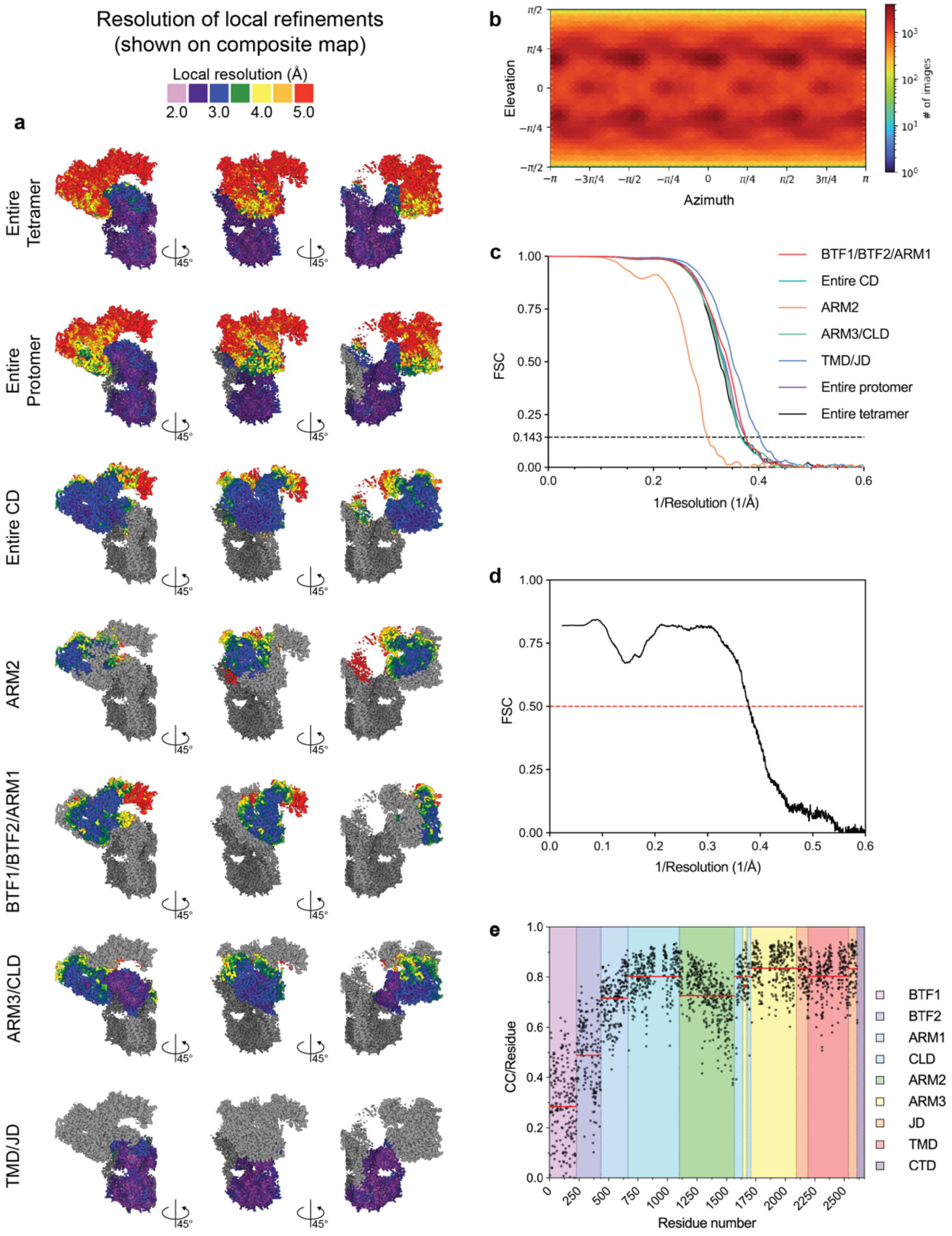

Supplementary Figure 9: Validation of the inhibited state. a, Local resolution plots at $FSC_{0.5}$ for consensus or local refinements depicted on the final density modified composite map. **b,** Angular distribution plot for the consensus refinement. **c,** Half map $FSC_{0.143}$ plots for all refinements. **d,** $FSC_{0.5}$ map versus model. **e,** Per-residue cross correlation (CC) for map-to-model comparison with domain demarcations and a red bar highlighting the average CC for each domain.

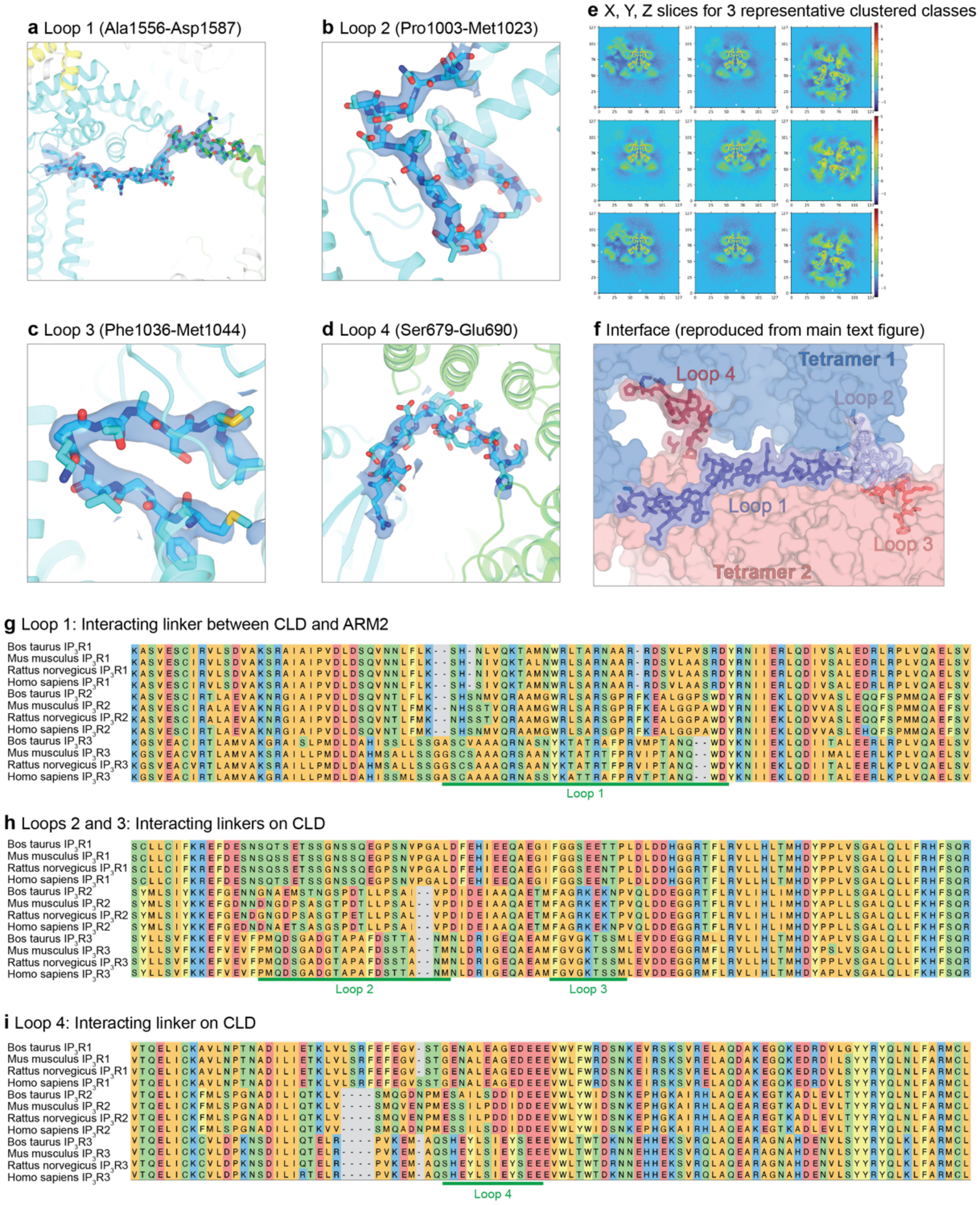

Supplementary Figure 10: Higher-order interactions between inhibited particles. a-d, Structures of inter-protomer interface in higher-order interactions. Loop 1 (a), loop 2 (b), loop 3 (c) and loop 4 (d) are depicted as sticks. Cryo-EM density shown as blue isosurface. **e,** Central slices through the density for representative classes of the clustered inhibited state showing strong density for an interaction with an adjacent protomer that extends outside of the refinement box. **f,** Model of the interacting loops between two tetramers (colored pink and blue) with the loops contributed from each shown as a similar color. **g,** Multiple sequence alignment for region between ARM2 and the CLD that contributes loop 1. **h,** Multiple sequence alignment for the region of the CLD contributing both loop 2 and loop 3. **i,** Multiple sequence alignment for the region of the CLD contributing loop 4.

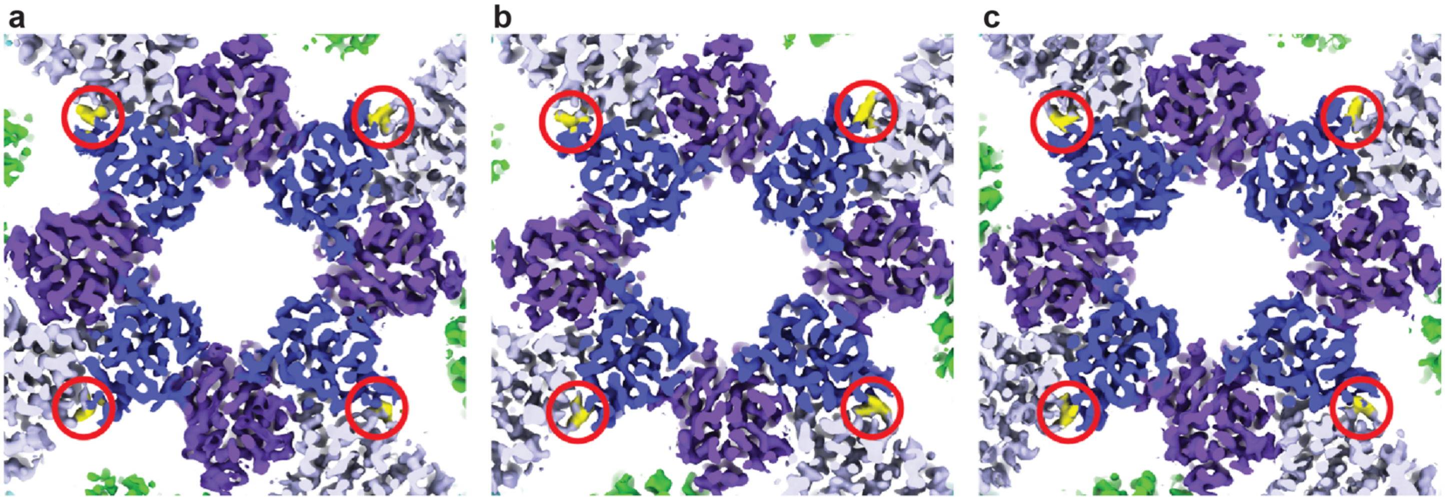

Supplementary Figure 11: IP_3 occupancy of the resting-to-preactivated transition states. a-c, Representative cryo-EM densities for the asymmetric 3:1 ARM2 extended:ARM2 retracted (a), 2:2 ARM2 extended:ARM2 retracted (b) and 1:3 ARM2 extended:ARM2 retracted (c) states that belong to the resting-to-reactivated transition ensemble. The density is colored by domain (purple for BTF1, blue for BTF2, light blue for ARM1, green for ARM2). Three-lobed density corresponding to IP_3 is colored yellow and circled in red.

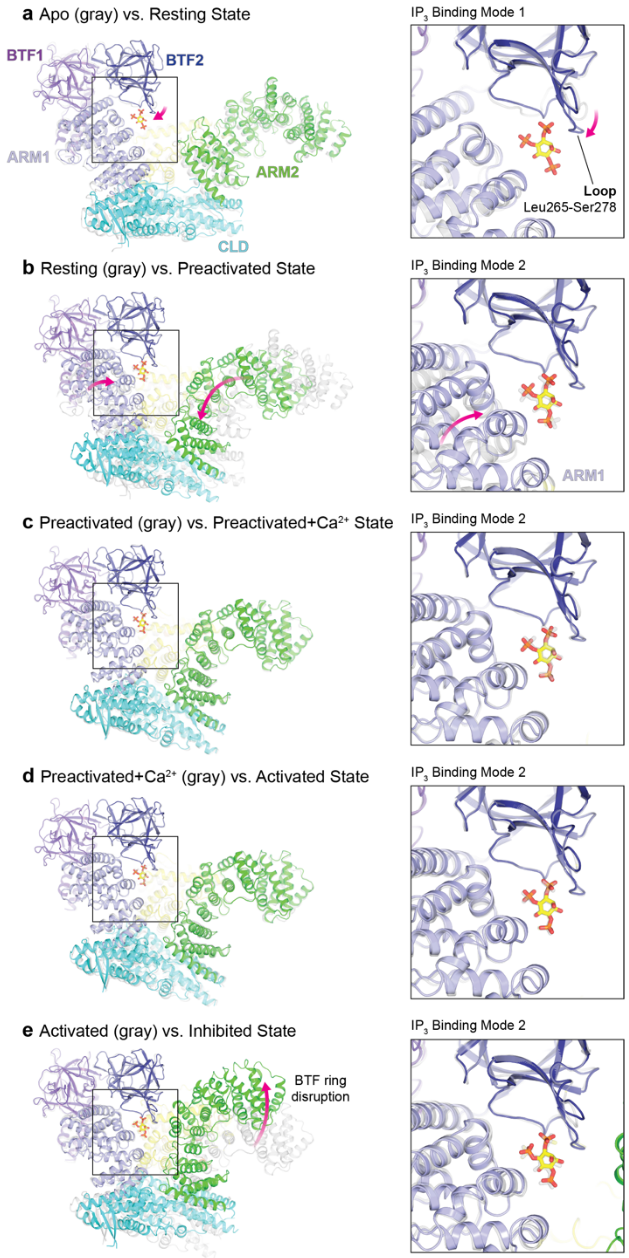

Supplementary Figure 12: IP$_3$ is accommodated in the binding pocket via two modes. a-e, Overlay of the entire CD (left) and IP$_3$-binding pocket (right) for the comparisons between ligand-free (apo) and resting states (a), resting and preactivated states (b), preactivated and preactivated+Ca^{2+} states (c), preactivated+Ca^{2+} and activated states (d), and activated and inhibited (e) states.

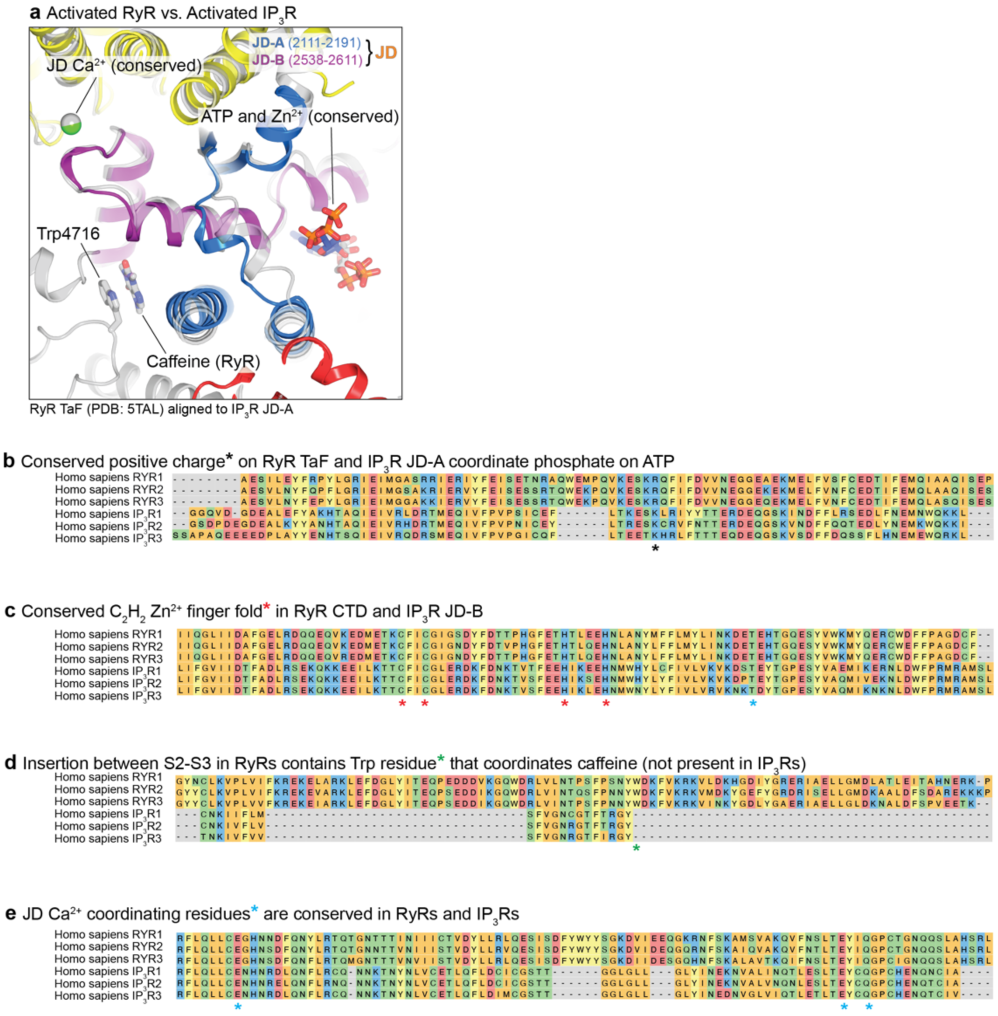

Supplementary Figure 13: Superposition of the JD ligand binding sites in IP$_3$Rs and RyRs. a, Overlay of rabbit RyR1 (gray) and hIP$_3$R3 (colored) in their activated states highlighting the conserved JD Ca^{2+}, ATP, and Zn^{2+} binding sites. The RyR caffeine binding site is not observed in hIP$_3$R3. The JD, composed of JD-A (blue) and JD-B (purple) are homologous to the thumb-and-forefinger (TaF) and C-terminal domains (CTD) in RyRs. The models were aligned by the TaF and JD-A. **b-e,** Multiple sequence alignments for IP$_3$Rs and RyRs highlighting conserved positive charge that coordinates phosphate on ATP (b, black asterisk), conserved C_2H_2 Zn^{2+} finger fold that coordinates Zn^{2+} ion (c, red asterisks), insertion in RyRs that forms caffeine binding site via a trytophan (d, green asterisk), and conserved JD Ca^{2+} coordinating residues (e, cyan asterisks).

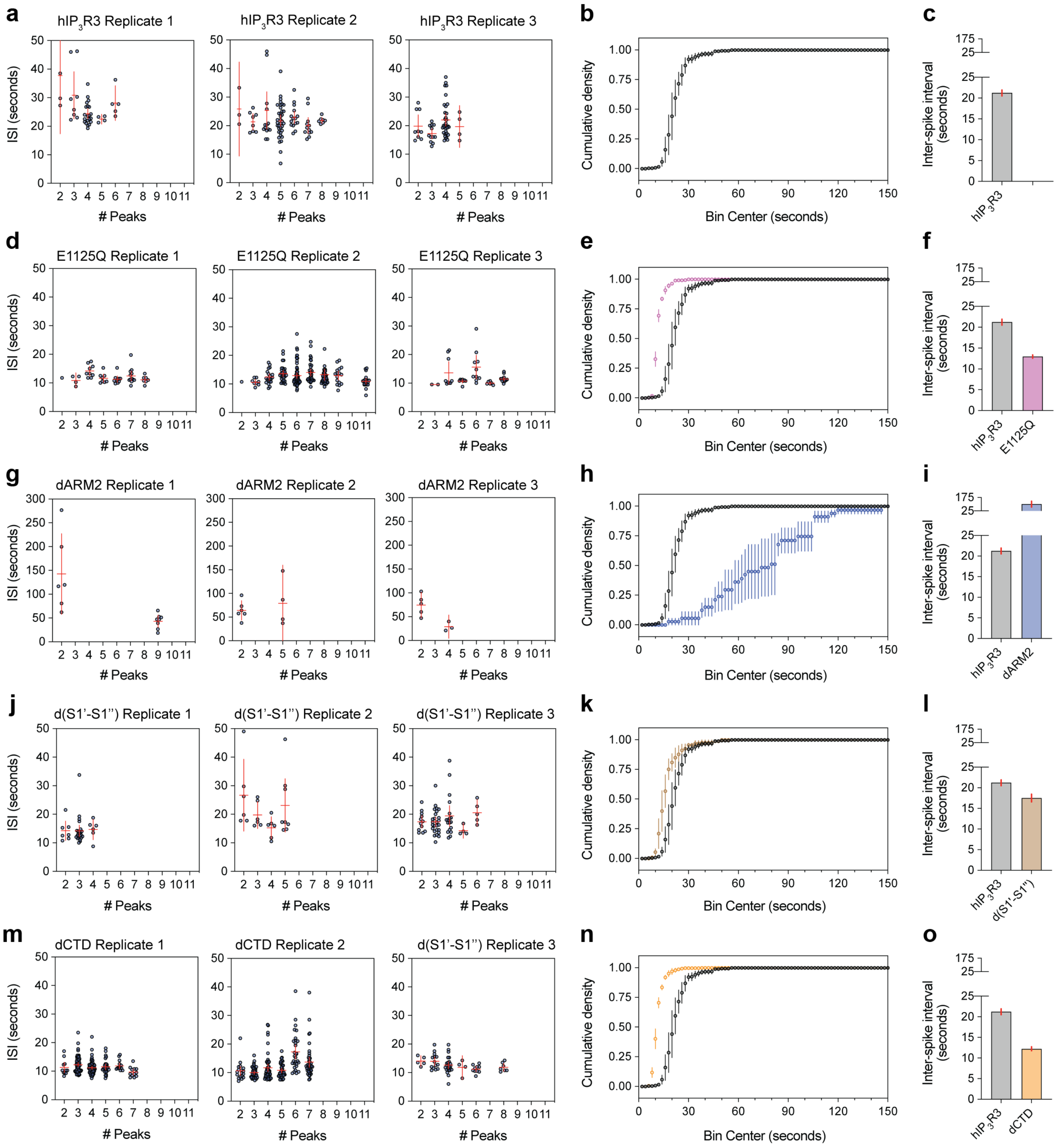
a
hIP3R3 Replicate 1
hIP3R3 Replicate 2
hIP3R3 Replicate 3
ISI (seconds)
Peaks
b
Cumulative density
Bin Center (seconds)
c
Inter-spike interval (seconds)
hIP3R3
d
E1125Q Replicate 1
E1125Q Replicate 2
E1125Q Replicate 3
e
f
E1125Q
g
dARM2 Replicate 1
dARM2 Replicate 2
dARM2 Replicate 3
h
i
dARM2
j
d(S1'-S1'') Replicate 1
d(S1'-S1'') Replicate 2
d(S1'-S1'') Replicate 3
k
l
d(S1'-S1'')
m
dCTD Replicate 1
dCTD Replicate 2
d(S1'-S1'') Replicate 3
n
o
dCTD

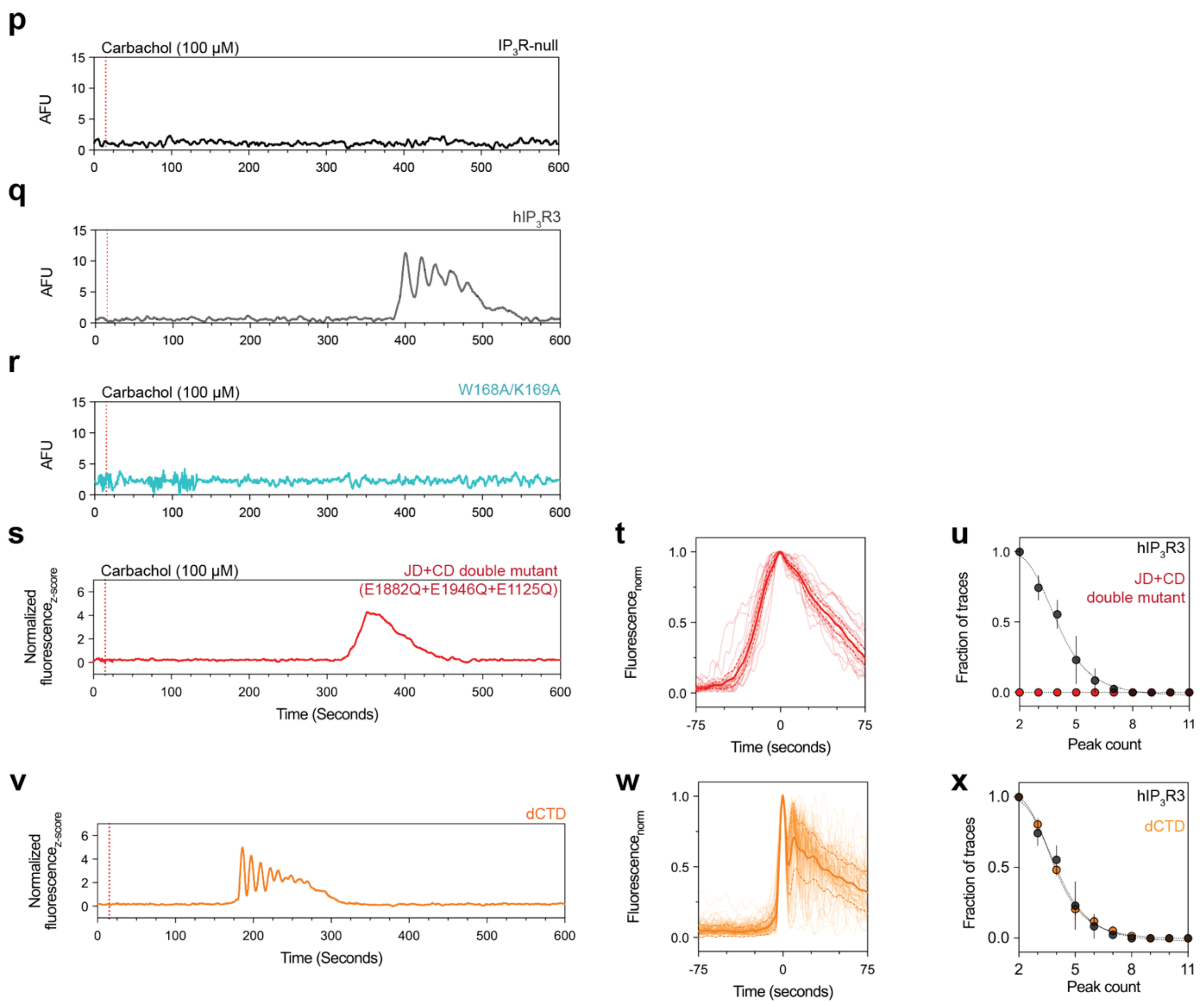

Supplementary Figure 14: Ca^{2+} imaging data analysis. a, d, g, j, m, Whisker plot of inter-spike intervals for N=3 biologically independent samples sorted by the minimum number of identifiable peaks observed in traces from which their values were obtained for hIP_3R3 (a), E1125Q (d), dARM2 (g), d(S1'-S1'') (j), and dCTD (m) mutants. Error bars correspond to 95% confidence interval around the mean. **b, e, h, k, n,** Cumulative density plot for inter-spike intervals from N=3 biologically independent samples for hIP3R3 (b), E1125Q (e), dARM2 (h), d(S1'-S1'') (k), and dCTD (n) mutants. Error bars correspond to 95% confidence interval around the mean. **c, f, i, l, o,** Bar plots depicting the mean inter-spike interval values for hIP_3R3 (c), E1125Q (f), dARM2 (i), d(S1'-S1'') (l), and dCTD (o) mutants. Error bars correspond to 95% confidence interval around the S.E.M. **p-r,** Representative filtered, and baseline adjusted traces for IP_3R-null cell line (p), cells overexpressing hIP_3R3 (q) and W168A/K169A (r). N=3 biologically independent samples. **s, v,** Representative z-score normalized Cal-520-AM fluorescence trace recorded from cells expressing E1882Q+E1946Q+E1125Q (s) and dCTD (v) mutants in an IP_3R-null background, following stimulation by carbachol**.** N=3 biologically independent samples **t, w,** Aligned first peak of every oscillatory trace normalized to 1 for E1882Q+E1946Q+E1125Q (t) and dCTD (w) mutants. **u, x,** Distribution of peak counts for all

oscillatory traces for E1882Q+E1946Q+E1125Q (u) and dCTD (x) mutants. Individual points represent mean and error bars represent S.E.M. Source data are provided as a Source Data file.

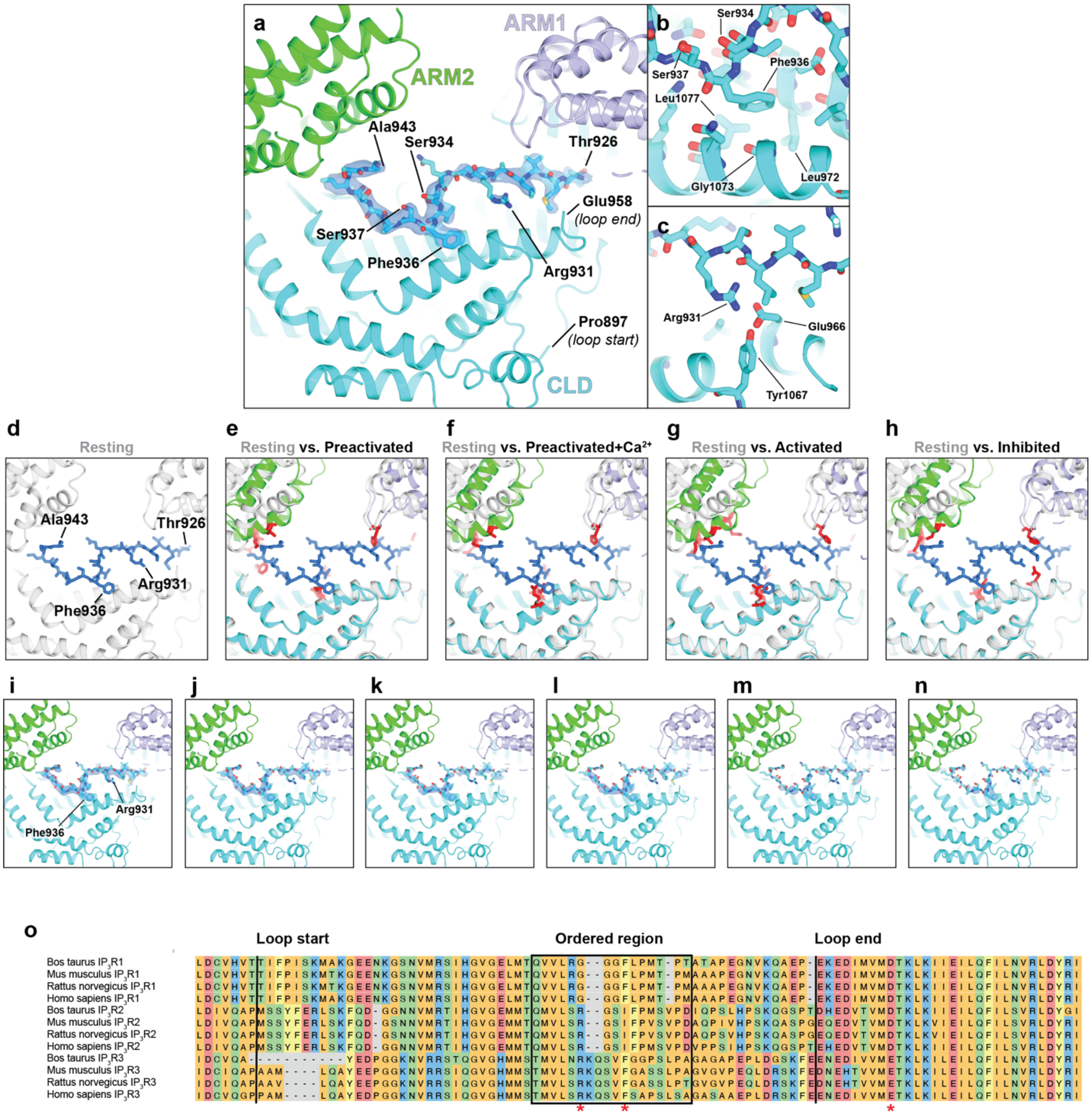

Supplementary Figure 15: Wedge loop prevents ARM2 retraction to stabilize the resting state. **a,** The wedge loop occupies a cavity between ARM1, ARM2 and the CLD in the resting state. Ordered residues within the wedge loop are depicted as sticks. Cryo-EM density for the wedge loop is shown as a blue isosurface. **b-c,** Interactions that stabilize the wedge loop at Phe936 (b) and Arg931 (c). **d-h,** Model of wedge loop docked from the resting state with alignment by the CLD. All residues with atoms closer than 2.7 Å are shown as red sticks for resting (d), preactivated (e), preactivated+Ca^{2+} (f), activated (g), and inhibited (h) states. **i-n,** Expanded 3DVA trajectory of the ARM2 extended position shown as blue isosurface reveals progressive disordering of the wedge loop from panels i to n. **o,** Multiple sequence alignment of the wedge loop. Arg931, Phe936 and Glu966 are highlighted with red asterisks.

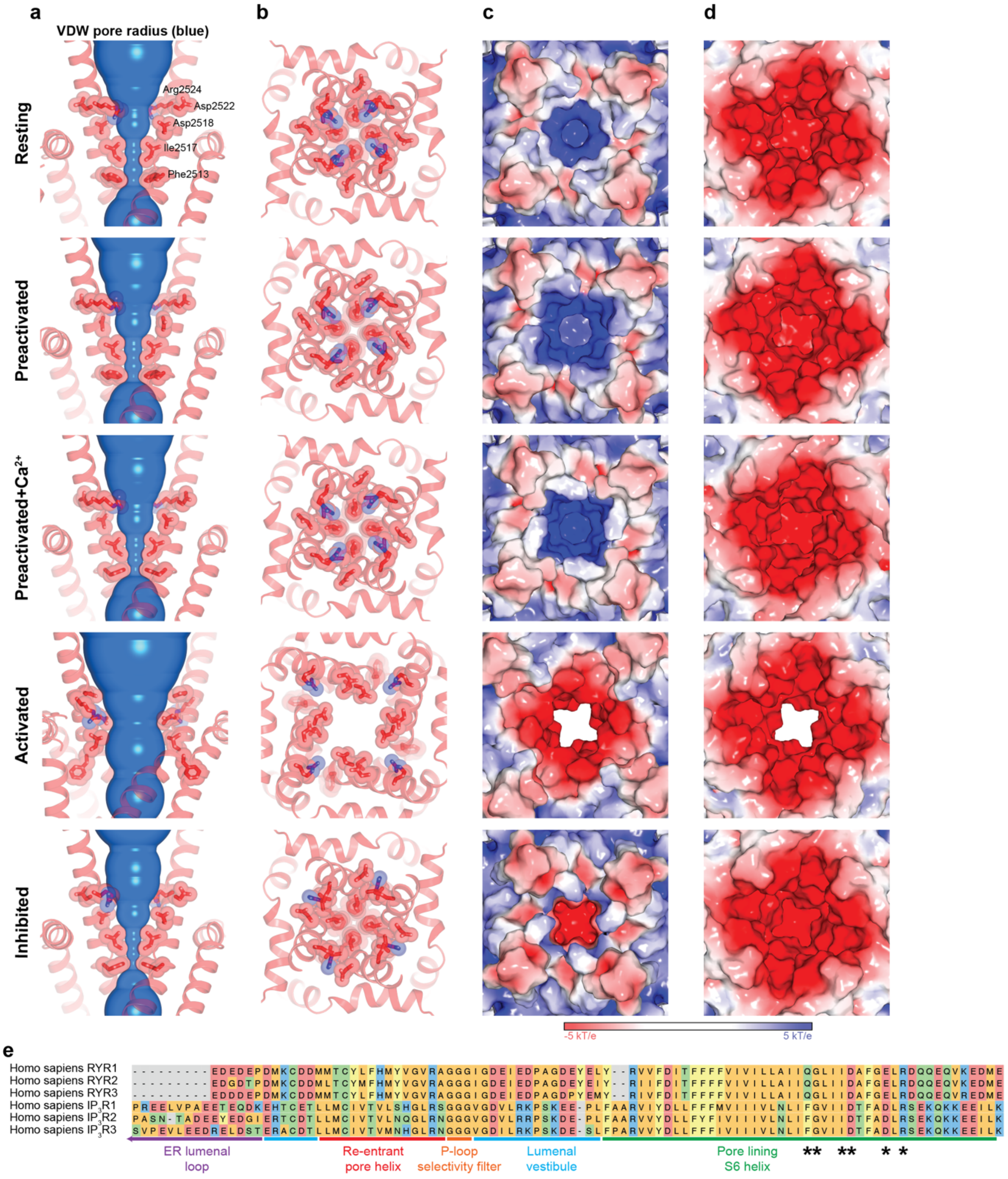

Supplementary Figure 16: Conformation and electrostatics of the pore. a-b, Structure of the pore viewed from the side (a) and cytosol (b) with front and rear protomers removed for clarity in a. Phe2513, Ile2517, Asp2518, Asp2522, Arg2524 shown as sticks and surfaces. **c-d,** Surface depiction viewed from the cytosol (c) and lumen (d) colored by electrostatic surface potential in the cytosolic portion of the pore. **e,** Multiple sequence alignment comparing luminal vestibule, pore helix, selectivity filter, and S6 helices in human IP_3Rs and RyRs.

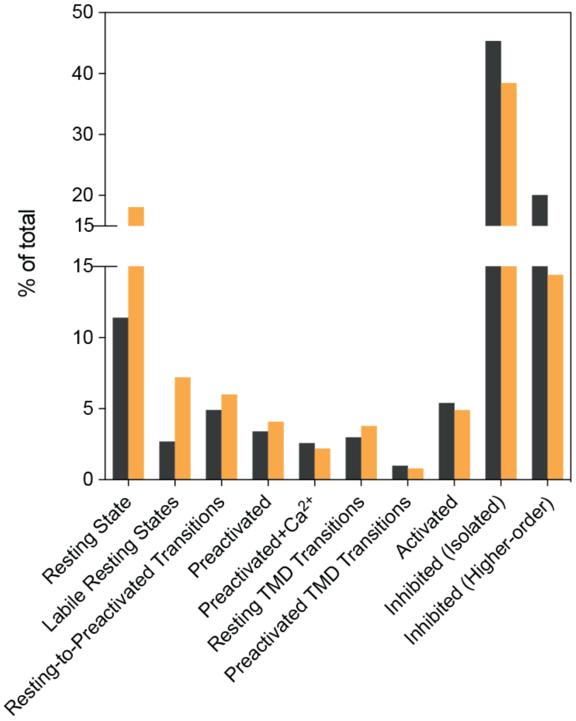

Supplementary Figure 17: Further analysis of the Ca^{2+}-dependent conformational landscape of hIP_3R3. Comparison of the relative abundances of all states from two grids prepared at 100 nM Ca^{2+}, shown here as black (602,609 particles) and gold (219,465 particles). Source data are provided as a Source Data file.

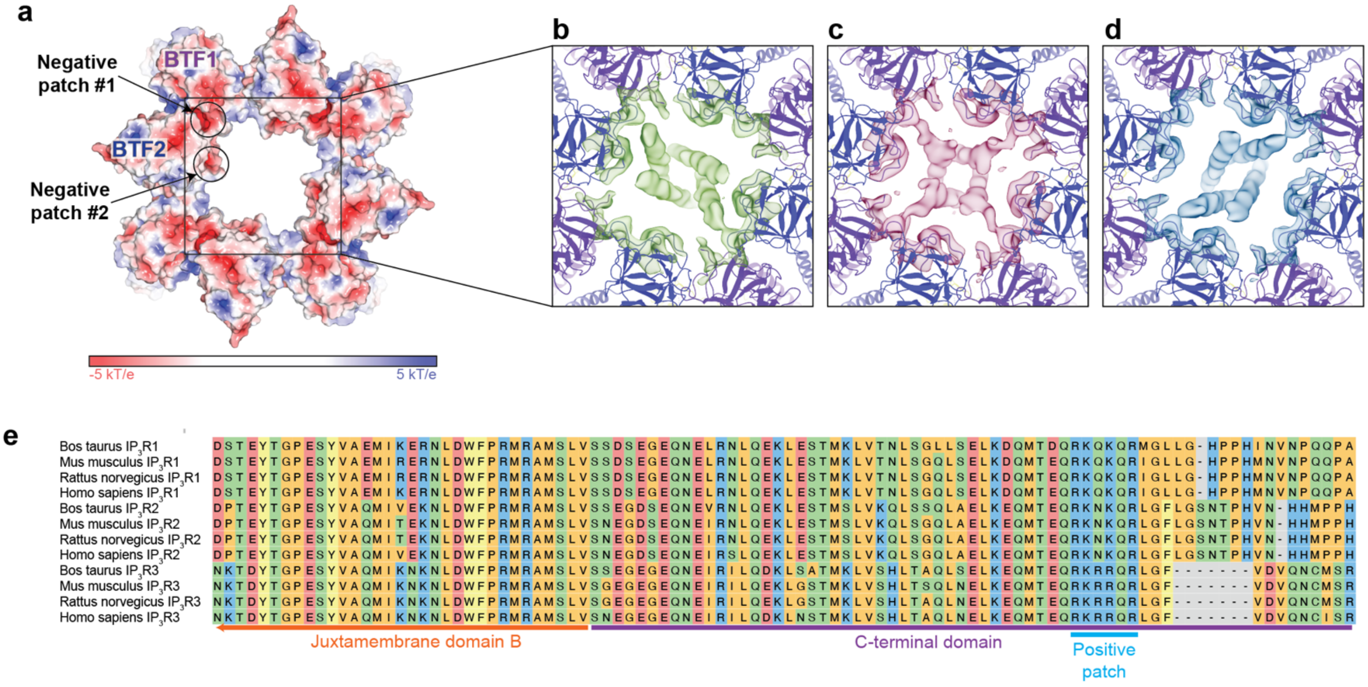

Supplementary Figure 18: The CTD dynamically interacts with two electronegative patches on the BTF ring. a, Electrostatic surface potential of the BTF ring of the activated state. Two electronegative patches highlighted with circles on a single protomer. **b-d,** Cryo-EM density shown on the activated state model of the BTF ring for three conformations. **e,** Multiple sequence alignment shows that a stretch of positive residues on the CTD is conserved.

Free [Ca^{2+}]	**1 nM**	**10 nM**	**100 nM (#1)**	**100 nM (#2)**	**1 μM**	**10 μM**
Total Micrographs	637	2150	6126	4312	1327	3136
Microscope	FEI Krios	FEI Krios	FEI Krios	FEI Krios	FEI Krios	FEI Krios
Voltage (kV)	300	300	300	300	300	300
Magnification	29,000	29,000	29,000	29,000	29,000	29,000
Dose Rate (e^-/pixel/sec)	15	15	15	15	15	15
Detector	Gatan K3	Gatan K3	Gatan K3	Gatan K3	Gatan K3	Gatan K3
Pixel Size (Å)	0.826	0.826	0.826	0.826	0.826	0.826
Exposure Time (sec)	3	3	3	3	3	3
Frames	60	60	60	60	60	60
Exposure (e^-/Å^2)	66	66	66	66	66	66
Defocus Range (μm)	-0.5 to -2.5	-0.5 to -2.5	-0.5 to -2.5	-0.5 to -2.5	-0.5 to -2.5	-0.5 to -2.5
Average Particles Per Micrograph Picked	119	125	135	77	99	105
Average Particles Per Micrograph Kept	89	95	100	52	79	81
Particles Kept (%)	75%	76%	74%	68%	80%	77%
Particles Kept (total)	56,681	203,838	614,301	225,190	108,079	254,044

Supplementary Table 1: Acquisition parameters for each data set.

	Resting	Preactivated	Preactivated+Ca^{2+}	Activated	Inhibited
EMDB ID	EMD- 41323	EMD- 41347	EMD- 41348	EMD- 41349	EMD- 41350
Final Pixel Size (Å)	0.629	0.629	0.629	0.629	0.629
Box Size (pixels)	512	512	512	512	512
**Map Validation:**					
Resolution (Half-map FSC = 0.143; Å) Consensus / Protomer / CD / TMD+JD / CLD+ARM3 / BTF1+BTF2+ARM1 / ARM2	2.7 / 2.7 / 2.7 / 2.5 / 2.8 / 2.7 / 3.3	3.7 / 3.7 / 3.8 / 3.6 / 4.0 / 3.9 / 6.5	3.6 / 3.5 / 3.4 / 3.5 / 3.5 / 3.3 / 4.2	3.2 / 3.1 / 3.0 / 3.0 / 3.0 / 2.9 / 3.3	2.5 / 2.6 / 3.0 / 2.5 / 2.7 / 3.4 / 3.4
B-factor (Guinier plot) Consensus / Protomer / CD / TMD+JD / CLD+ARM3 / BTF1+BTF2+ARM1 / ARM2	81 / 84 / 88 / 81 / 91 / 89 / 120	68 / 93 / 121 / 115 / 125 / 147 / 802	67 / 80 / 89 / 117 / 109 / 100 / 146	80 / 85 / 86 / 98 / 95 / 86 / 125	80 / 90 / 122 / 105 / 105 / 179
Refinement Symmetry	C1	C1	C1	C1	C1
Total Symmetry Expanded Particles (Final Map)	767,556 (271,434 for ARM2)	186,210	123,416	228,188	3,668,312 (357,163 for BTF1+BTF2 +ARM1)
**PDB ID**	8TK8	8TKD	8TKE	8TKF	8TKG
Model Composition:					
Atoms (Non-hydrogen)	74,692	72,316	72,320	74,100	73,400
Chains	4	4	4	4	4
Protein Residues	9,072	8,996	8,996	9,088	8,840
Ions Ca2+ / Zn2+	0 / 4	0 / 4	4 / 4	4 / 4	8 / 4
Ligands ATP / IP_3 / Lipids	4 / 4 / 24	4 / 4 / 0	4 / 4 / 0	4 / 4 / 20	4 / 4/ 24
Waters	612	0	0	0	640
**Model Validation:**					
Map-to-Model (FSC = 0.5 for density-modified composite of local refinements)	2.79	3.79	3.67	3.16	2.60

Map-to-Model (CC) BTF1 / BTF2 / ARM1 / CLD / ARM2 / ARM3 / JD / TMD / CTD	0.82 / 0.79 / 0.78 / 0.76 / 0.76 / 0.77 / 0.79 / 0.76 / 0.54	0.75 / 0.73 / 0.72 / 0.75 / 0.74 / 0.75 / 0.75 / 0.73 / 0.66	0.82 / 0.82 / 0.81 / 0.81 / 0.80 / 0.78 / 0.79 / 0.76 / 0.64	0.74 / 0.73 / 0.74 / 0.74 / 0.75 / 0.74 / 0.72 / 0.71 / 0.56	0.28 / 0.49 / 0.72 / 0.80 / 0.73 / 0.83 / 0.83 / 0.80 / 0.70
Mean B-Factor (Å^2) Protein / Ligand / Water (NA if not present)	72.6 / 85.1 / 55.7	101.9 / 79.7 / NA	80.0 / 76.5 / NA	96.1 / 98.3 / NA	85.6 / 76.8 / 53.8
R.M.S. Deviations Bond Lengths (Å) / Bond Angles (°)	0.002 / 0.406	0.002 / 0. 467	0.002 / 0.454	0.002 / 0.421	0.002 / 0.483
MolProbity Score	1.12	1.37	1.29	1.18	1.34
CaBLAM outliers (%)	0.95	1.14	1.18	1.31	0.73
Clash Score	3.29	6.63	5.39	3.98	6.08
Rotamer Outliers (%)	0.66	0.25	0.1	0.44	0.24
Cβ Deviations	0	0	0	0	0
Ramachandran Plot (%) Favored / Allowed / Disallowed	98.09 / 1.91 / 0.00	98.20 / 1.76 / 0.05	98.58 / 1.42 / 0.00	98.04 / 1.96 / 0.00	87.98 / 2.02 / 0.00

Supplementary Table 2: Validation of major state models.

	Higher-Order Inhibited Symmetry Mate #1	Higher-Order Inhibited Symmetry Mate #2	Labile Resting #1	Labile Resting #2
EMDB ID	EMD-41366	EMD-41365	EMD- 41351	EMD- 41352
**Map Validation:**				
Consensus Resolution (Half-map FSC = 0.143; Å)	3.2	3.3	3.5	3.6
Refinement Symmetry	C1	C1	C1	C1
Total Symmetry Expanded Particles (Final Map)	85,139	88,082	91,326	155,671
**PDB ID**	8TLA	8TL9	8TKH	8TKI
Model Composition:				
Atoms (Non-hydrogen)	65,076	65,069	79,972	79,972
Chains	4	4	4	4
Protein Residues	8,015	8,014	9,072	9,072
Ions Ca2+ / Zn2+	4 / 4	4 / 4	0 / 4	0 / 4
Ligands ATP / IP_3	4/ 4	4 / 4	4 / 4	4 /4
Waters	0	0	0	0
**Model Validation:**				
Map-to-Model (FSC = 0.5 for density-modified composite of local refinements)	3.40	3.83	3.61	3.88
Mean B-Factor ($Å^2$) Protein / Ligand / Water (NA if not present)	134.3 / 149.0	137.4 / 159.0	79.5 / 59.8	54.1 / 45.7
R.M.S. deviations Bond Lengths (Å) / Bond Angles (°)	0.002 / 0.517	0.002 / 0.500	0.002 / 0.443	0.002 / 0.472
MolProbity Score	1.42	1.39	1.26	1.35
CaBLAM outliers (%)	0.76	0.81	0.72	1
Clash Score	7.69	7.09	4.91	6.31

Rotamer Outliers (%)	0.12	0.36	0.1	0.15
Cβ Deviations	0	0	0	0
Ramachandran Plot (%) Favored / Allowed / Disallowed	98.56 / 1.42 / 0.03	98.51 / 1.48 / 0.01	98.75 / 1.25 / 0.00	98.39 / 1.61 / 0.00

Supplementary Table 3: Validation of minor state models.

	Fig. 5a (3/1 ext/ret)	Fig. 5a (2/2 ext/ret)	Fig. 5a (1/3 ext/ret)	Fig. 5b	Fig. 5c	Fig. 5d	Fig. 5e	Fig. 5f	Fig. 5g
EMDB ID	EMD-41324	EMD-41325	EMD-41326	EMD-41327	EMD-41328	EMD-41329	EMD-41330	EMD-41331	EMD-41332
Resolution (Half-map FSC = 0.143; Å)	4	4	3.9	4	3.7	4.1	3.8	3.7	4
B-factor (Guinier Plot)	52	24	17	48	69	90	97	82	52
Low-Pass Filter (Å)	5	5	5	5	5	5	5	5	5
Refinement Symmetry	C1	C1	C1	C1	C1	C1	C1	C1	C1
Total Symmetry Expanded Particles (Final Map)	83,868	31,541	25,729	21,877	62,262	87,974	100,749	81,743	24,087
**Note** Maps shown in Figure 5a are representative and not exhaustive i.e. multiple classes produced these conformations that were later combined into the resting-to-preactivated transition ensemble.									

Supplementary Table 4: Validation of cryo-EM density presented in Figure 5.

	Preactivated TMD Transition (~C2)	Preactivated TMD Transition (~C4)	Resting TMD Transition (~C2)	Resting TMD Transition (~C4)
EMDB ID	EMD- 41339	EMD- 41340	EMD- 41344	EMD- 41345
Resolution (Half-map FSC = 0.143; Å)	3.7	3.6	3.6	3.7
B-factor (Guinier Plot)	33	44	35	46
Low-Pass Filter (Å) Overview / Zoomed	4 / 7	4 / 7	4 / 7	4 / 7
Refinement Symmetry	C1	C1	C1	C1
Total Symmetry Expanded Particles (Final Map)	34,006	66,872	63,075	121,444

Supplementary Table 5: Validation of cryo-EM density presented in Fig. 7 and Supplementary Fig. 8.

	Panel i	Panel j	Panel k	Panel l	Panel m	Panel n
EMDB ID	EMD-41334	EMD-41338	EMD-41335	EMD-41337	EMD-41336	EMD-41333
Resolution (Half-map FSC = 0.143; Å)	3.8	3.1	3.1	3.5	3.2	3
B-factor (Guinier Plot)	103	58	81	105	88	67
Refinement Symmetry	C1	C1	C1	C1	C1	C1
Total Symmetry Expanded Particles (Final Map)	195,763	49,313	176,457	194,283	186,458	89,264

Supplementary Table 6: Validation of cryo-EM density presented in Supplementary Fig. 15.

	1 nM	10 nM	100 nM (#1)	100 nM (#2)	1 µM	10 µM
Resting State	25248	54108	68808	39701	2296	1728
Labile Resting States	11416	13412	16435	15877	1605	3005
Resting TMD Transitions	3353	7663	18093	8247	2706	6070
Resting-to-Preactivated Transitions	4182	21176	29757	13212	1096	1252
Preactivated	2013	12804	20482	8929	870	1454
Preactivated (+Ca2+)	615	8896	15643	4792	520	388
Preactivated TMD Transitions	184	2051	6131	1862	437	222
Activated	565	8218	32272	10684	2978	2330
Inhibited (Clustered)	3322	24419	121239	31703	18529	47031
Inhibited (Isolated)	4988	48075	273749	84458	74422	185143

Supplementary Table 7: Raw particle counts for titration analysis.

Cell line/construct	**IP_3R-null**	**hIP_3R3**	**CD site mutant**	**JD site mutant**	**JD/CD sites double mutant**	**dARM2**	**dS1'-S1''**	**BTF ring single mutant**	**BTF ring double mutant**	**dCTD**
Tag (w/ Position at N or C terminus)	N/A	N-10xHis-EGFP	N-10xHis-EGFP	N-10xHis-EGFP	N-10xHis-EGFP	N-10xHis-EGFP	N-10xHis-EGFP	N-10xHis-EGFP	N-10xHis-EGFP	N-10xHis-EGFP
Linker Sequence (Tag-Linker-Gene)	N/A	LEVLFQGPSRV	LEVLFQGPSRV	LEVLFQGPSRV	LEVLFQGPSRV	LEVLFQGPSRV	LEVLFQGPSRV	LEVLFQGPSRV	LEVLFQGPSRV	LEVLFQGPSRV
Mutation	N/A	N/A	E1125Q	E1882Q, E1946Q	E1882Q, E1946Q, E1125Q	Δ(A1101-W1586)	Δ(D2252-I2302)	K169A	W168A, K169A	Stop codons at L2629x, N2630x
Mean inter-spike interval (ISI) (seconds)	NR	21.7	12.7	NR	NR	117.1	17.6	- NR	- NR	12.1
Lower 95% CI of mean (ISI) (seconds)	NR	20.9	12.4	NR	- NR	81	16.5	NR	NR	11.7
Upper 95% CI of mean (ISI) (seconds)	NR	22.5	13	NR	NR	153.2	18.7	NR	NR	12.4
Median no. of peaks/cell	NR	4	6	NR	NR	1	3	NR	NR	4
Normalized fluorescence/ second at half maxima ± SEM (n=3)	- NR	0.103 ± 0.015	0.143 ± 0.011	0.028 ± 0.006	0.033 ± 0.003	0.173 ± 0.010	0.130 ± 0.019	0.026 ± 0.001	NR	0.188 ± 0.013
NR - No response.										

Supplementary Table 8: Cal-520-AM calcium imaging analysis.

	CTD Green	CTD Blue	CTD Red
EMDB ID	EMD- 41341	EMD- 41342	EMD- 41343
Resolution (Half-map FSC = 0.143; Å)	5.25	5.22	5.55
B-factor (Guinier Plot)	305	289.5	388.5
Refinement Symmetry	C1	C1	C1
Total Symmetry Expanded Particles (Final Map)	68,453	61,216	73,883

Supplementary Table 9: Validation of cryo-EM density presented in Supplementary Fig. 18.

Mask	Protomer	Assembled Protomer? (Y/N)	Resolution
TMD/JD	1	N	3.1
TMD/JD	2	N	3
TMD/JD	3	Y	3
TMD/JD	4	Y	3
ARM3/CLD	1	N	3.8
ARM3/CLD	2	N	3.8
ARM3/CLD	3	Y	3.3
ARM3/CLD	4	Y	3.4
Entire CD	1	N	4.5
Entire CD	2	N	5.2
Entire CD	3	Y	3.4
Entire CD	4	Y	3.9
BTF1/BTF2/ARM1	1	N	7
BTF1/BTF2/ARM1	2	N	7.1
BTF1/BTF2/ARM1	3	Y	6.8
BTF1/BTF2/ARM1	4	Y	7
ARM2	1	N	7.1
ARM2	2	N	7.2
ARM2	3	Y	6.1
ARM2	4	Y	5.4
Entire Protomer	1	N	3.2
Entire Protomer	2	N	3.2
Entire Protomer	3	Y	3.2
Entire Protomer	4	Y	3.2
Consensus (Tetramer)	All	N/A	3.2

Supplementary Table 10: Local refinements comprising higher-order symmetry mate #1.

Mask	Protomer	Assembled Protomer? (Y/N)	Resolution
TMD/JD	1	N	3
TMD/JD	2	N	3
TMD/JD	3	Y	3
TMD/JD	4	Y	3
ARM3/CLD	1	N	3.8
ARM3/CLD	2	N	3.8
ARM3/CLD	3	Y	3.4
ARM3/CLD	4	Y	3.3
Entire CD	1	N	4.9
Entire CD	2	N	5.5
Entire CD	3	Y	4
Entire CD	4	Y	3.4
BTF1/BTF2/ARM1	1	N	7.2
BTF1/BTF2/ARM1	2	N	7
BTF1/BTF2/ARM1	3	Y	6.9
BTF1/BTF2/ARM1	4	Y	6.5
ARM2	1	N	7.2
ARM2	2	N	7.1
ARM2	3	Y	6.2
ARM2	4	Y	3.6
Entire Protomer	1	N	3.2
Entire Protomer	2	N	3.2
Entire Protomer	3	Y	3.1
Entire Protomer	4	Y	3.2
Consensus (Tetramer)	All	N/A	3.3

Supplementary Table 11: Local refinements comprising higher-order symmetry mate #2.

Oligo description	Mutagenesis method	Oligo Sequence (5'-3')
hIP3R3-E1125Q Forward Primer	Single-primer reactions	ACCCACAGCTGTGACTTCTCCACCAT GGTC
hIP3R3-E1125Q Reverse Primer	Single-primer reactions	GACCATGGTGGAGAAGTCACAGCTGT GGGT
hIP3R3-E1882Q Forward Primer	Single-primer reactions	GTTGTGGTTCTGACACAGCAGCTGCA GAAAGC
hIP3R3-E1882Q Reverse Primer	Single-primer reactions	GCTTTCTGCAGCTGCTGTGTCAGAAC CACAAC
hIP3R3-E1946Q Forward Primer	Single-primer reactions	TGGCAGTACTGAGTGAGGGTCTCCAA GGTC
hIP3R3-E1946Q Reverse Primer	Single-primer reactions	GACCTTGGAGACCCTCACTCAGTACT GCCA
hIP3R3-dS1'-S1" N-Terminal Forward Primer	NEBuilder® HiFi DNA Assembly	CGGCTACTTTTTCTCTGTCACAGAATG AAAATTTTTC
hIP3R3-dS1'-S1" N-Terminal Reverse Primer	NEBuilder® HiFi DNA Assembly	GCCCGATGCCCATGTAAGGGTAGAAG AAGG
hIP3R3-dS1'-S1" C-Terminal Forward Primer	NEBuilder® HiFi DNA Assembly	CCCTTACATGGGCATCGGGCCCACAC TC
hIP3R3-dS1'-S1" C-Terminal Reverse Primer	NEBuilder® HiFi DNA Assembly	TGACAGAGAAAAAGTAGCCGAAGATG ACGGTTTGTC
hIP3R3-dCTD N-Terminal Forward Primer	NEBuilder® HiFi DNA Assembly	CGGCTACTTTTTCTCTGTCACAGAATG AAAATTTTTC
hIP3R3-dCTD N-Terminal Reverse Primer	NEBuilder® HiFi DNA Assembly	CATTGCTGACCATGACTCGAGATGGA CC
hIP3R3-dCTD C-Terminal Forward Primer	NEBuilder® HiFi DNA Assembly	TCGAGTCATGGTCAGCAATGAGGGCG AG
hIP3R3-dCTD C-Terminal Reverse Primer	NEBuilder® HiFi DNA Assembly	TGACAGAGAAAAAGTAGCCGAAGATG ACG
hIP3R3-dARM2 N-Terminal Forward Primer	NEBuilder® HiFi DNA Assembly	CGGCTACTTTTTCTCTGTCACAGAATG AAAATTTTTC
hIP3R3-dARM2 N-Terminal Reverse Primer	NEBuilder® HiFi DNA Assembly	TCTTGTAGTCTGAGATCAGCAGCTGA AC

hIP3R3-dARM2 C-Terminal Forward Primer	NEBuilder® HiFi DNA Assembly	GCTGATCTCAGACTACAAGAACATCATTGAG
hIP3R3-dARM2 C-Terminal Reverse Primer	NEBuilder® HiFi DNA Assembly	TGACAGAGAAAAAGTAGCCGAAGATGAC
hIP3R3-K169A N-Terminal Forward Primer	NEBuilder® HiFi DNA Assembly	CGGCTACTTTTTCTCTGTCACAGAATGAAAATTTTTC
hIP3R3-K169A N-Terminal Reverse Primer	NEBuilder® HiFi DNA Assembly	TCCGCAGGGCCCAGAAGGGCTGGATGAAG
hIP3R3-K169A C-Terminal Forward Primer	NEBuilder® HiFi DNA Assembly	GCCCTTCTGGGCCCTGCGGAGCAACGGG
hIP3R3-K169A C-Terminal Reverse Primer	NEBuilder® HiFi DNA Assembly	TGACAGAGAAAAAGTAGCCGAAGATGACGGTTTGTCACATG
hIP3R3-W168A,K169A N-Terminal Forward Primer	NEBuilder® HiFi DNA Assembly	CGGCTACTTTTTCTCTGTCACAGAATGAAAATTTTTC
hIP3R3-W168A,K169A N-Terminal Reverse Primer	NEBuilder® HiFi DNA Assembly	CCAGCCCTTCGCCGCCCTGCGGAGCAACGGGGAC
hIP3R3-W168A,K169A C-Terminal Forward Primer	NEBuilder® HiFi DNA Assembly	TCGAGTCATGGTCAGCAATGAGGGCGAG
hIP3R3-W168A,K169A C-Terminal Reverse Primer	NEBuilder® HiFi DNA Assembly	GCAGGGCGGCGAAGGGCTGGATGAAGAG

Supplementary Table 12: Oligos used for the generation of hIP_3R3 mutants described in this study.
