# Supplementary material for: Structural titration reveals Ca2+-dependent conformational landscape of the IP3 receptor
Source: Nat Commun. 2023 Oct 28;14:6897. doi: 10.1038/s41467-023-42707-3 (PMC10613215; doi:10.1038/s41467-023-42707-3)
Supplement: Supplementary file 3 — Description of Additional Supplementary Files [file 41467_2023_42707_MOESM3_ESM.pdf]

Description of Additional Supplementary Files

File name: Supplementary Movie 1

Description: 6 modes of 3DVA for resting state.

File name: Supplementary Movie 2

Description: 6 modes of 3DVA for preactivated state.

File name: Supplementary Movie 3

Description: 6 modes of 3DVA for preactivated+Ca^{2+} state.

File name: Supplementary Movie 4

Description: 6 modes of 3DVA for activated state.

File name: Supplementary Movie 5

Description: 6 modes of 3DVA for resting-to-preactivated transitions.

File name: Supplementary Movie 6

Description: 6 modes of 3DVA for resting TMD transitions.

File name: Supplementary Movie 7

Description: 6 modes of 3DVA for preactivated TMD transitions.

File name: Supplementary Movie 8

Description: 6 modes of 3DVA for inhibited state.

File name: Supplementary Movie 9

Description: Preactivated+Ca^{2+} to activated morph, view of TMD and JD from cytosol.

File name: Supplementary Movie 10

Description: Preactivated+Ca^{2+} to activated morph, view of TMD and JD from membrane plane.

File name: Supplementary Movie 11

Description: Preactivated+Ca^{2+} to activated morph, view of TMD and JD for a single chain from membrane plane.
