# Supplementary material for: Structural titration reveals Ca2+-dependent conformational landscape of the IP3 receptor
Source: Nat Commun. 2023 Oct 28;14:6897. doi: 10.1038/s41467-023-42707-3 (PMC10613215; doi:10.1038/s41467-023-42707-3)
Supplement: Supplementary file 15 — Reporting Summary [file 41467_2023_42707_MOESM15_ESM.pdf]

nature portfolio

Corresponding author(s): Hite, Richard K

Last updated by author(s): Oct 6, 2023

Reporting Summary

Nature Portfolio wishes to improve the reproducibility of the work that we publish. This form provides structure for consistency and transparency in reporting. For further information on Nature Portfolio policies, see our Editorial Policies and the Editorial Policy Checklist.

Statistics

For all statistical analyses, confirm that the following items are present in the figure legend, table legend, main text, or Methods section.

n/a	Confirmed	
☐	☒	The exact sample size (*n*) for each experimental group/condition, given as a discrete number and unit of measurement
☐	☒	A statement on whether measurements were taken from distinct samples or whether the same sample was measured repeatedly
☒	☐	The statistical test(s) used AND whether they are one- or two-sided<br>*Only common tests should be described solely by name; describe more complex techniques in the Methods section.*
☒	☐	A description of all covariates tested
☒	☐	A description of any assumptions or corrections, such as tests of normality and adjustment for multiple comparisons
☐	☒	A full description of the statistical parameters including central tendency (e.g. means) or other basic estimates (e.g. regression coefficient) AND variation (e.g. standard deviation) or associated estimates of uncertainty (e.g. confidence intervals)
☒	☐	For null hypothesis testing, the test statistic (e.g. *F*, *t*, *r*) with confidence intervals, effect sizes, degrees of freedom and *P* value noted<br>*Give P values as exact values whenever suitable.*
☒	☐	For Bayesian analysis, information on the choice of priors and Markov chain Monte Carlo settings
☒	☐	For hierarchical and complex designs, identification of the appropriate level for tests and full reporting of outcomes
☒	☐	Estimates of effect sizes (e.g. Cohen's *d*, Pearson's *r*), indicating how they were calculated

Our web collection on statistics for biologists contains articles on many of the points above.

Software and code

Policy information about availability of computer code

Data collection	SerialEM, CryoSparc Live v3.3.1, Zen 2.3
Data analysis	CryoSparc v3.3.1, PyEM, Relion v3.1.3, Phenix v1.20.1-4487, Coot 0.9.6, PyMol (Schrodinger, LLC. 2010. The PyMOL Molecular Graphics System, Version 2.5.3), ChimeraX 1.5, Chimera 1.15, MATLAB 9.12.0.1884302 (R2022a), ImageJ, Fiji, GraphPad Prism 9, customized MATLAB scripts (https://github.com/vinay-sapuru/hIP3R3.git)

For manuscripts utilizing custom algorithms or software that are central to the research but not yet described in published literature, software must be made available to editors and reviewers. We strongly encourage code deposition in a community repository (e.g. GitHub). See the Nature Portfolio guidelines for submitting code & software for further information.

Data

Policy information about availability of data

All manuscripts must include a data availability statement. This statement should provide the following information, where applicable:

- Accession codes, unique identifiers, or web links for publicly available datasets
- A description of any restrictions on data availability
- For clinical datasets or third party data, please ensure that the statement adheres to our policy

The Cryo-EM data and atomic coordinates generated in this study have been deposited in the Electron Microscopy Data Bank and Protein Data Bank under accession codes EMD-41323 [https://www.ebi.ac.uk/emdb/EMD-41323] and 8TK8 [https://www.rcsb.org/structure/8TK8] for the Resting state structure,

EMD-41347 [https://www.ebi.ac.uk/emdb/EMD-41347] and 8TKD [https://www.rcsb.org/structure/8TKD] for the Preactivated state structure, EMD-41348 [https://www.ebi.ac.uk/emdb/EMD-41348] and 8TKE [https://www.rcsb.org/structure/8TKE] for the Preactivated+Ca2+ state structure, EMD-41349 [https://www.ebi.ac.uk/emdb/EMD-41349] and 8TKF [https://www.rcsb.org/structure/8TKF] for the Activated state structure, EMD-41350 [https://www.ebi.ac.uk/emdb/EMD-41350] and 8TKG [https://www.rcsb.org/structure/8TKG] for the Inhibited state structure, EMD-41351 [https://www.ebi.ac.uk/emdb/EMD-41351] and 8TKH [https://www.rcsb.org/structure/8TKH] for the Labile resting state 1 structure, EMD-41352 [https://www.ebi.ac.uk/emdb/EMD-41352] and 8TKI [https://www.rcsb.org/structure/8TKI] for the Labile resting state 2 structure, EMD-41366 [https://www.ebi.ac.uk/emdb/EMD-41366] and 8TLA [https://www.rcsb.org/structure/8TLA] for the Higher-order inhibited state 1 structure and EMD-41365 [https://www.ebi.ac.uk/emdb/EMD-41365] and 8TL9 [https://www.rcsb.org/structure/8TL9] for the Higher-order inhibited state 2 structure. Cryo-EM data generated in this study have been deposited in the Electron Microscopy Data Bank under accession code EMD-41324 [https://www.ebi.ac.uk/emdb/EMD-41324], EMD-41325 [https://www.ebi.ac.uk/emdb/EMD-41325] and EMD-41326 [https://www.ebi.ac.uk/emdb/EMD-41326] for the Resting-to-Preactivated transition states, EMD-41327 [https://www.ebi.ac.uk/emdb/EMD-41327], EMD-41328 [https://www.ebi.ac.uk/emdb/EMD-41328], EMD-41329 [https://www.ebi.ac.uk/emdb/EMD-41329], EMD-41330 [https://www.ebi.ac.uk/emdb/EMD-41330], EMD-41331 [https://www.ebi.ac.uk/emdb/EMD-41331] and EMD-41332 [https://www.ebi.ac.uk/emdb/EMD-41332] for the ARM2 retractions states, EMD-41333 [https://www.ebi.ac.uk/emdb/EMD-41333], EMD-41334 [https://www.ebi.ac.uk/emdb/EMD-41334], EMD-41335 [https://www.ebi.ac.uk/emdb/EMD-41335], EMD-41336 [https://www.ebi.ac.uk/emdb/EMD-41336], EMD-41337 [https://www.ebi.ac.uk/emdb/EMD-41337] and EMD-41338 [https://www.ebi.ac.uk/emdb/EMD-41338] for the Wedge loop progression states, EMD-41339 [https://www.ebi.ac.uk/emdb/EMD-41339] for the ~C2 Preactivated TMD Transition, EMD-41340 [https://www.ebi.ac.uk/emdb/EMD-41340] for the ~C4 Preactivated TMD Transition, EMD-41341 [https://www.ebi.ac.uk/emdb/EMD-41341], EMD-41342 [https://www.ebi.ac.uk/emdb/EMD-41342] and EMD-41343 [https://www.ebi.ac.uk/emdb/EMD-41343] for the Activated CTD states, EMD-41344 [https://www.ebi.ac.uk/emdb/EMD-41344] for the ~C2 Resting TMD Transition, and EMD-41345 [https://www.ebi.ac.uk/emdb/EMD-41345] for the ~C4 Resting TMD Transition. The atomic coordinates of previously published structures used in this study at available at the Protein Data Bank under accession codes 3JAV [https://doi.org/10.2210/pdb3JAV/pdb], 6MU2 [https://doi.org/10.2210/pdb6MU2/pdb], 7LHF [https://doi.org/10.2210/pdb7LHF/pdb] and 7LHE [https://doi.org/10.2210/pdb7LHE/pdb] for rat Type 1 IP3R, 6DQJ [https://doi.org/10.2210/pdb6DQJ/pdb] and 6UQK [https://doi.org/10.2210/pdb6UQK/pdb] for human Type 3 IP3R in a resting state, 6DQS [https://doi.org/10.2210/pdb6DQS/pdb], 6DQZ [https://doi.org/10.2210/pdb6DQZ/pdb], and 6DR0 [https://doi.org/10.2210/pdb6DR0/pdb] for human type 3 IP3R resting-to-preactivated transition states, 6DQV [https://doi.org/10.2210/pdb6DQV/pdb], 7T3P [https://doi.org/10.2210/pdb7T3P/pdb], 7T3Q [https://doi.org/10.2210/pdb7T3Q/pdb], and 7T3R [https://doi.org/10.2210/pdb7T3R/pdb] for human type 3 IP3R in preactivated states, 7T3T [https://doi.org/10.2210/pdb7T3T/pdb] for human type 3 IP3R in an activated state, 6DRC [https://doi.org/10.2210/pdb6DRC/pdb], 6DR2 [https://doi.org/10.2210/pdb6DR2/pdb], 6DRA [https://doi.org/10.2210/pdb6DRA/pdb], and 7T3U [https://doi.org/10.2210/pdb7T3U/pdb] for human type 3 IP3R in inhibited states, crystal structures of the IP3 binding site (1N4K [https://doi.org/10.2210/pdb1N4K/pdb], 3T8S [https://doi.org/10.2210/pdb3T8S/pdb], and 3UJ0 [https://doi.org/10.2210/pdb3UJ0/pdb] for fragments containing the IP3-binding domain of rat type 1 IP3R, and 5TAP [https://doi.org/10.2210/pdb5TAP/pdb] for caffeine- and ATP-bound rabbit RyR1. Source data are provided with this paper. Plasmids are available upon request.

Human research participants

Policy information about studies involving human research participants and Sex and Gender in Research.

Reporting on sex and gender	N/A
Population characteristics	N/A
Recruitment	N/A
Ethics oversight	N/A

Note that full information on the approval of the study protocol must also be provided in the manuscript.

Field-specific reporting

Please select the one below that is the best fit for your research. If you are not sure, read the appropriate sections before making your selection.

☒ Life sciences ☐ Behavioural & social sciences ☐ Ecological, evolutionary & environmental sciences

For a reference copy of the document with all sections, see nature.com/documents/nr-reporting-summary-flat.pdf

Life sciences study design

All studies must disclose on these points even when the disclosure is negative.

Sample size	Cryo-EM: Data was collected for each Ca2+ condition as deemed sufficient to populate various states for classification and dependent on the limitations of the microscope during acquisition.<br><br>Ca2+ Imaging: No statistical methods were used to determine the required sample size. Sample size was arbitrarily selected based on the time required to collect data. The number of cells responding to stimulation by carbachol were variable for each experiment, and therefore only responding cells were analyzed. The sample size was limited to the number of responding cells in aggregate for each replicate.
Data exclusions	Cryo-EM images with high drift, bad ice or contamination were removed. False-positive particle selections were removed by heterogeneous classification.
Replication	Cryo-EM: Images were collected from two grids prepared with 100 nM Ca2+ and one grid each prepared with 1 nM, 10 nM, 1 µM and 10 µM Ca2+. The two grids at 100 nM support successful replication of the ligand addition strategy.

	Ca2+ Imaging: Movies (Cal-520-AM imaging) were collected on different days but under the same conditions for all three biological replicates. Three biological replicates for each construct were utilized for calculation of confidence intervals. In addition to the biological replicates, each condition was replicated at least one time.
Randomization	Cryo-EM: Particles were randomized following extraction to avoid bias during particle classification.<br><br>Ca2+ Imaging: Fluorescence spectra of Cal-520-AM and mVenus overlap, and variations in fluorescence background from mVenus-hIP3R3 constructs across cells required manual adjustment of thresholds for each Cal-520-AM fluorescence trace to accurately identify peaks. Additionally, the presence of oscillatory spikes prevented automatic baseline adjustment in our MATLAB scripts, requiring manual intervention. Due to these reasons, randomization was not performed.
Blinding	Cryo-EM: All classification was performed with combined data in a blinded fashion prior to assignment to the specific Ca2+ concentrations from which the data were derived.<br><br>Ca2+ Imaging: These experiments were not blinded, but peak assignments were performed by a software algorithm with minimal human intervention.

Reporting for specific materials, systems and methods

We require information from authors about some types of materials, experimental systems and methods used in many studies. Here, indicate whether each material, system or method listed is relevant to your study. If you are not sure if a list item applies to your research, read the appropriate section before selecting a response.

Materials & experimental systems

n/a	Involved in the study
☒	☐ Antibodies
☐	☒ Eukaryotic cell lines
☒	☐ Palaeontology and archaeology
☒	☐ Animals and other organisms
☒	☐ Clinical data
☒	☐ Dual use research of concern

Methods

n/a	Involved in the study
☒	☐ ChIP-seq
☒	☐ Flow cytometry
☒	☐ MRI-based neuroimaging

Eukaryotic cell lines

Policy information about cell lines and Sex and Gender in Research

Cell line source(s)	Human Embryonic kidney (IP3R null: HEK-3KO; https://www.kerafast.com/productgroup/703/ip3r-expressing-hek-293-cell-lines?ProductID=4481)<br>HEK293S GnTl- (ATCC CRL-3022)<br>Sf9 cells (Expression system:94-001F)
Authentication	Cells were not authenticated as they were purchased less than one year ago.
Mycoplasma contamination	Cell lines were not tested for mycoplasma contamination as they were purchased less than one year ago.
Commonly misidentified lines<br>(See ICLAC register)	None were used in this study.
